# Primal-dual interior point QP-free algorithm for nonlinear constrained optimization

**DOI:** 10.1186/s13660-017-1500-2

**Published:** 2017-09-29

**Authors:** Jinbao Jian, Hanjun Zeng, Guodong Ma, Zhibin Zhu

**Affiliations:** 1 School of Mathematics and Statistics, Guangxi Colleges and Universities Key Laboratory of Complex System Optimization and Big Data Processing, Yulin Normal University, Yulin, China; 2 College of Mathematics and Information Science, Guangxi University, Nanning, China; 3 College of Mathematics and Computing Science, Guilin University of Electronic Technology, Guilin, China

**Keywords:** 90C30, 49M37, 65K05, inequality and equality constraints, optimization, primal-dual interior method, working set, global and superlinear convergence

## Abstract

In this paper, a class of nonlinear constrained optimization problems with both inequality and equality constraints is discussed. Based on a simple and effective penalty parameter and the idea of primal-dual interior point methods, a QP-free algorithm for solving the discussed problems is presented. At each iteration, the algorithm needs to solve two or three reduced systems of linear equations with a common coefficient matrix, where a slightly new working set technique for judging the active set is used to construct the coefficient matrix, and the positive definiteness restriction on the Lagrangian Hessian estimate is relaxed. Under reasonable conditions, the proposed algorithm is globally and superlinearly convergent. During the numerical experiments, by modifying the technique in Section 5 of (SIAM J. Optim. 14(1): 173-199, 2003), we introduce a slightly new computation measure for the Lagrangian Hessian estimate based on second order derivative information, which can satisfy the associated assumptions. Then, the proposed algorithm is tested and compared on 59 typical test problems, which shows that the proposed algorithm is promising.

## Introduction

In this paper, we consider nonlinear constrained optimization problems with inequality and equality constraints

1 $$ \mathrm{(P)}\quad \min \ f(x), \quad \mbox{s.t.} \quad g_{i}(x)= 0, \quad i\in I^{\ell } ; \qquad g_{j}(x) \leq 0, \quad j \in I^{\imath }, $$

where $I^{\ell }=\{1,2,\ldots,m_{\ell }\}, I^{\imath }=\{m_{\ell }+1,m _{\ell }+2,\ldots,m_{\ell }+m_{\imath }\}$, the functions *f* and $g_{j}: R^{n}\rightarrow R$. It is known that the nonlinear equality constraints are difficult to be dealt with in designing algorithms for (P), especially, in designing the *methods of feasible directions* (MFD). In 1976, Mayne and Polak [2] proposed a simple scheme to convert (P) to a sequence of inequality smoothing constrained optimization

2 $$ {\mathrm{(P}}_{\rho })\quad \operatorname{min} \ f_{\rho }(x):=f(x)-\rho \sum_{j\in I^{\ell }}g_{j}(x), \quad \mbox{s.t.}\quad g_{j}(x)\leq 0, \quad j\in I^{\ell }\cup I^{\imath }, $$

where $\rho >0$ is a penalty parameter. Under suitable *constraint qualifications* (CQ), e.g., linear independence, it has been shown that $(\mathrm{P}_{\rho })$ is equivalent to (P) when *ρ* is large enough. So, based on $(\mathrm{P}_{\rho })$, one can study and present effective algorithms for the original problem (P), e.g., Refs. [1, 3–6].

In addition, with the help of inequality constrained non-smoothing optimization

$$ \min \ f(x)+\sum_{j\in I^{\ell }}c_{j} \bigl\vert g_{j}(x) \bigr\vert , \quad \mbox{s.t.} \quad g _{j}(x)\leq 0,\quad j\in I^{\ell }\cup I^{\imath }, $$

one can also design an algorithm for solving the original problem (P), e.g., [7], where $c_{j}>0$ is the penalty parameter that needs to be updated.

It is known that the *sequential quadratic programming* (SQP) method is one of the efficient methods for constrained optimization due to its fast convergence, and it has been widely studied by many authors, see Refs. [8–17]. However, the *quadratic program* (QP) subproblems solved in the SQP methods may be inconsistent, and the computational cost for the QPs is high. Therefore, motivated by the KKT condition of the QPs and/or the quasi-Newton method, QP-free methods are put forward, in which the QPs are replaced by suitable *systems of linear equations* (SLEs), see Refs. [18–26].

Now we review briefly the study on the *primal-dual interior point* (PDIP) QP-free algorithms associated with our work. First, for problem (P) with no equality constraints, i.e., $I^{\ell }=\emptyset $, in 1987, Panier et al. [22] presented a QP-free algorithm denoted by PTH, at iterate *k*, two SLEs are solved to yield a master search direction. Then a *least squares problem* (LSP) needs to be solved to avoid the so-called Maratos effect [27]. However, the SLEs solved in [22] may become ill-conditioned, and the PTH algorithm may be instable. Furthermore, the initial point must lie on the strict interior of the feasible set, and an additional assumption that ‘the number of stationary points is finite’ is used to ensure the global convergence. Later, under the assumption that the multiplier approximation sequence remains bounded, the PTH algorithm was improved by Gao et al. [3] by solving an extra SLE. The PTH algorithm was also improved by Qi and Qi [23], Zhu [26] and Cai [28].

To improve the PTH algorithm [22], by using the idea of PDIP and choosing different barrier parameters for each constraint, Bakthiari and Tits [18] proposed a new PDIP QP-free algorithm. The algorithm can start from a feasible point at the boundary of the feasible set, and it possesses global convergence without both the additional assumption of isolatedness of the stationary points and the positive definite restriction on matrix $H_{k}$. Almost at the same time, Tits et al. [1] extended and improved the PTH algorithm to problem (P) with both inequality and equality constraints. The algorithm [1] possesses two remarkable characters. One is that a new and simple rule to update the penalty parameter *ρ* in (P_*ρ*_) is derived, the other is that, same as in [18], the uniformly positive definite restriction on the Lagrangian Hessian estimate is relaxed.

More recently, for inequality constrained optimization, Jian et al. [21] proposed a strongly sub-feasible primal-dual quasi interior-point algorithm with superlinear convergence, where the initial point can be chosen arbitrarily, the number of feasible constraints is nondecreasing, and the iteration points all enter into the interior of the feasible region after finite iterations; a new kind of working set was introduced, which further reduced the computational cost; the uniformly positive definite restriction on the sequence $\{H_{k}\}$ was relaxed; at each iteration, only two or three SLEs with the same coefficient matrix needed to be solved.

However, there are still some problems worthy of research on the PDIP-type algorithms [1, 18, 22]. First, the coefficient matrix of the Karush-Kuhn-Tucker (KKT) system of the LSP is not the same as the two previous SLEs, and this further increases the computational cost. Second, the coefficient matrices of the SLEs include all the constraints and their gradients, and this leads to a large increase in the scale of the SLEs. Third, the global convergence of the two algorithms [1, 18] relies on an additional assumption that the stationary points are finite or isolated.

On the other hand, to design more effective algorithms with small computational cost for solving constrained optimization, Facchinei et al. [29] first introduced the active set identifying technique (also called working set technique). And then this technique has been popularized and applied in many works, e.g., [17, 24, 25, 30, 31]. Particularly, the algorithm [30] needs to solve four SLEs at each iteration.

The goal of this paper is to improve and extend the algorithms [18, 21] to nonlinear constrained optimization (P) and, at the same time, to overcome the three problems mentioned above. As a result, by means of problem (P_*ρ*_), we propose a PDIP-type algorithm for problem (P). Compared with the previous PDIP-type algorithms, the proposed algorithm possesses the following features.

- A slightly new identifying technique for the active set different from [17, 25] is introduced. The multiplier yielded at the previous iteration is used to compute the working set, and no additional computational cost is needed, so the computational cost is expected to be reduced.
- At each iteration, to yield the search directions, only two or three SLEs with the same coefficient matrix need to be solved. Furthermore, the coefficient matrix has smaller scale than the ones in [1, 18, 22].
- For a strict interior point $x^{k}$ of the feasible set of (P_*ρ*_), the iteration at $x^{k}$ is well defined without any other constraint qualification (CQ).
- Under suitable CQ and assumptions including a relaxed positive definite restriction on the Lagrangian Hessian estimate $H_{k}$, *but without the isolatedness of the stationary points*, the proposed algorithm is globally and superlinearly convergent.
- A slightly new computation technique for $H_{k}$ based on second order derivative information is introduced, which is a modification of the one in [1], Section 5.1, and satisfies the relaxed positive definite restriction.

Throughout this paper, for simplicity, denote vector $(x^{T},y^{T},z ^{T},\ldots )^{T}$ by $(x,y,z,\ldots )$ for column vectors $x, y$ and *z*, and $\Vert \cdot \Vert $ denotes the Euclidean norm.

## Construction of algorithm

To analyze our algorithm, the following notations are used:

$$\begin{aligned}& I= I^{\ell }\cup I^{\imath }, \qquad \hat{e}= \bigl(1, \dots,1(m_{\ell }\mbox{th}),0, \dots,0 \bigl((m_{\ell }+m_{\imath })\mbox{th} \bigr) \bigr)^{T}, \\& X= \bigl\{ x\in R^{n}: g_{i}(x)=0, i\in I^{\ell }; g_{j}(x)\leq 0, j \in I^{\imath } \bigr\} , \qquad e_{J}=(1,\dots,1)^{T}\in R^{\vert J \vert }, \\& \tilde{X}= \bigl\{ x\in R^{n}: g_{j}(x)\leq 0, j\in I \bigr\} , \qquad \tilde{X_{0}}= \bigl\{ x\in R^{n}: g_{j}(x)< 0, j\in I \bigr\} , \\& I^{\ell }(x)= \bigl\{ j\in I^{\ell }: g_{j}(x)=0 \bigr\} , \qquad I^{\imath }(x)= \bigl\{ j \in I^{\imath }: g_{j}(x)=0 \bigr\} ,\qquad I(x)=I^{\ell }(x)\cup I^{\imath }(x), \\& g_{\ell }(x)= \bigl(g_{j}(x),j\in I^{\ell } \bigr),\qquad g_{\imath }(x)= \bigl(g_{j}(x),j \in I^{\imath } \bigr),\qquad g(x)= \bigl(g_{j}(x), j\in I \bigr), \\& g_{J}(x)= \bigl(g_{j}(x),j\in J\subset I \bigr),\qquad \nabla g_{J}(x)= \bigl(\nabla g_{j}(x), j\in J \bigr), \\& g_{j}^{k}=g_{j} \bigl(x^{k} \bigr), \qquad g_{J}^{k}=g_{J} \bigl(x^{k} \bigr), \qquad \nabla g_{j}^{k}= \nabla g_{j} \bigl(x^{k} \bigr),\qquad \nabla g_{j}^{k^{T}}= \bigl( \nabla g_{j}^{k} \bigr)^{T}. \end{aligned}$$

First, the following basic hypothesis is necessary.

**H1**: The inner set $\tilde{X_{0}}$ is nonempty, and the functions *f* and $g_{j}$ ($j\in I$) are all continuously differentiable.

### Remark 1

Note that if there exists a point belonging to the set *X̃*, namely, $\hat{x}\in \tilde{X}$, and the active constraint gradient vectors $\{ \nabla g_{j}(\hat{x}), j\in I(\hat{x})\}$ are linearly independent, then one can yield a point $x^{0}\in \tilde{X_{0}}$ by simple computation, e.g., execute line search on *g* starting with *x̂* along direction $\hat{d}=-\hat{N}(\hat{N} ^{T}\hat{N})^{-1}e$, where $\hat{N}=\nabla g_{I(\hat{x})}(\hat{x})$ and $e=(1,\ldots,1)^{T}$.

Before proposing our algorithm, we give a proposition to show the equivalences between (P) and $(\mathrm{P}_{\rho })$.

### Proposition 1

*If* $(x,\lambda )$ *is a KKT pair for problem* $(\mathrm{P}_{\rho})$ *and* $g_{\ell }(x)=0$, *then* $(x,\lambda_{\rho })$ *with multiplier* $\lambda_{\rho }=\lambda -\rho \hat{e}$ *is a KKT pair for the original problem* (P).

Based on Proposition 1, it is known that if one can construct an effective algorithm for problem $(\mathrm{P}_{\rho})$ and adjust parameter *ρ* to force the iterate to asymptotically satisfy $g_{\ell }(x)=0$, then the solution to (P) can be yielded.

Now, refer to [29] and [24], we introduce optimal identification functions Φ and *δ* as follows:

3 $$ \Phi (x,\lambda )=\left ( \textstyle\begin{array}{c} \nabla_{x}L (x,\lambda ) \\ g_{\ell }(x) \\ \min \{-g_{\imath }(x), \lambda_{\imath }\}\end{array}\displaystyle \right ), \qquad \delta (x, \lambda )= \bigl\Vert \Phi (x,\lambda ) \bigr\Vert ^{r}, $$

where $\lambda = (\lambda_{\ell },\lambda_{\imath })$, parameter $r\in (0,1)$, and the Lagrangian function

4 $$ L(x,\lambda )=f(x)+\sum_{j\in I} \lambda_{j}g_{j}(x). $$

It is clear that $(x^{*},\lambda )$ is a KKT pair of (P) if and only if $\delta (x^{*},\lambda )=0$. Particularly, from [29] or/and [24], Definition 4.1, Theorems 4.1, 4.2 and 4.3, one can see that $\{j\in I: g_{j}(x)+\delta (x,\lambda )\geq 0\}$ is an exact identification set for active constrain set $I(x^{*})$ if $(x,\lambda )$ converges to a KKT pair $(x^{*},\lambda ')$ of problem (P), and the Mangasarian-Fromovotz constraint qualification (MFCQ) and the second order sufficient conditions are satisfied at $(x^{*}, \lambda ')$.

In this paper, similarly to the techniques in [21, 30], for the current iterate $x^{k}\in \tilde{X} _{0}$, we yield the corresponding multiplier vector $\lambda^{k}= ( \lambda^{k}_{\ell },\lambda^{k}_{\imath })$ in (3)-(4) as follows:

5 $$ \lambda^{0}=z^{0}, \qquad \lambda^{k}=\bar{\lambda }^{k-1}-\rho_{k-1} \hat{e}, \quad k>0, $$

where $z^{0}>0$, and $(\bar{\lambda }^{k-1},\rho_{k-1})$ is computed in the previous iteration $(k-1)$th. Then, similarly to [29], we structure our working set by

6 $$ I^{\imath }_{k}= \bigl\{ j\in I^{\imath }: g_{j} \bigl(x^{k} \bigr)+\delta \bigl(x^{k}, \lambda^{k} \bigr)\geq 0 \bigr\} ,\qquad I_{k}= I^{\ell } \cup I^{\imath }_{k}. $$

The reason why one does not compute $I^{\ell }_{k}$ as $I^{\imath } _{k}$ is to force $g_{\ell }(x^{k})\rightarrow 0$, see the analysis of Theorem 1 in Section 3. The set $I^{\imath }_{k}$ equals the exact active set $I^{\imath }(x^{*})$ when $(x^{k},\lambda ^{k})$ is sufficiently close to a KKT pair $(x^{*},\lambda ')$ of (P) and the second order sufficient conditions as well as the MFCQ hold at $(x^{*},\lambda ')$. This important property allows us to construct the direction finding subproblems only considering the constraints in the working set $I_{k}$.

Taking into account that the iterates always execute within the feasible set *X̃*, let us consider the first order condition of optimality (KKT condition) for problem (P$_{\rho_{k}}$) nearby the current iterate $x^{k}$:

$$\begin{aligned} \nabla f_{\rho_{k}}(x)+\sum_{j\in I_{k}} \lambda_{j}\nabla g _{j}(x)=0, \qquad \lambda_{j} g_{j}(x)=0, \quad j\in I_{k}, \lambda_{I_{k}} \geq 0. \end{aligned}$$

Furthermore, if we ignore the non-negativity request ‘$\lambda_{I_{k}} \geq 0$’ and simultaneously introduce a suitable perturbation $((1-\zeta_{k})\nabla f_{\rho_{k}}(x^{k}),\mu^{k})\in R^{(n+\vert I_{k} \vert )}$ in the right-hand side of the above system, then it can be reduced as a system of nonlinear equations with variables $(x, \lambda_{I_{k}})$

7 $$ \left ( \textstyle\begin{array}{c} \nabla f_{\rho_{k}}(x)+\sum_{j\in I_{k}}\lambda_{j}\nabla g _{j}(x) \\ \lambda_{j} g_{j}(x), j\in I_{k} \end{array}\displaystyle \right ) = \left ( \textstyle\begin{array}{c} (1-\zeta_{k})\nabla f_{\rho_{k}}(x^{k}) \\ \mu^{k} \end{array}\displaystyle \right ) . $$

Applying the Newton method to system (7) starting with the current iterate $(x^{k}, \lambda^{k}_{I_{k}})$, it yields a SLE as follows:

8 $$ \left ( \textstyle\begin{array}{c@{\quad}c@{}} \nabla^{2}_{xx}L_{\rho_{k}}(x^{k},\lambda^{k}_{I_{k}}) & \nabla g_{{I_{k}}}(x^{k}) \\ \Lambda_{k}\nabla g_{I_{k}}(x^{k})^{T} & \operatorname{diag}(g^{k}_{I_{k}}) \end{array}\displaystyle \right ) \left ( \textstyle\begin{array}{c} x-x^{k} \\ \lambda_{I_{k}} \end{array}\displaystyle \right ) = \left ( \textstyle\begin{array}{c} -\zeta_{k}\nabla f_{\rho_{k}}(x^{k}) \\ \mu^{k} \end{array}\displaystyle \right ), $$

where diagonal matrix $\Lambda_{k}=\operatorname{diag}(\lambda^{k}_{I_{k}})$, and the Lagrangian Hessian

$$\begin{aligned} \nabla^{2}_{xx}L_{\rho_{k}} \bigl(x^{k}, \lambda^{k}_{I_{k}} \bigr)= \nabla^{2} f _{\rho_{k}} \bigl(x^{k} \bigr)+\sum_{j\in I_{k}} \lambda^{k}_{j}\nabla^{2} g _{j} \bigl(x^{k} \bigr). \end{aligned}$$

Subsequently, to make the coefficient matrix in SLE (8) possess nice property and low computational cost, we consider its optimization and modification as follows. First, replace the Lagrangian Hessian by a suitable approximate symmetric matrix $H_{k}$, and denote $x-x^{k}$ by direction *d*. Second, replace the diagonal matrix $\Lambda_{k}$ by positive diagonal matrix $Z_{k}=\operatorname{diag}(z^{k}_{I _{k}})$, where vector $z^{k}_{I_{k}}$ is an approximation of $\lambda^{k}$.

As a result, from system (8), the coefficient matrix and the form of the SLEs that need to be solved in our algorithm are as follows:

9 $$\begin{aligned}& V_{k}:= \left ( \textstyle\begin{array}{c@{\quad}c@{}} H_{k} & \nabla g_{I_{k}}(x^{k}) \\ Z_{k}\nabla g_{I_{k}}(x^{k})^{T} & \operatorname{diag}(g^{k}_{I_{k}}) \end{array}\displaystyle \right ), \end{aligned}$$

10 $$\begin{aligned}& \operatorname{SLE} \bigl(V_{k}; \zeta_{k}, \mu^{k} \bigr): \quad V_{k}\left ( \textstyle\begin{array}{c} d \\ \lambda_{I_{k}} \end{array}\displaystyle \right ) = \left ( \textstyle\begin{array}{c} -\zeta_{k}\nabla f_{\rho_{k}}(x^{k}) \\ \mu^{k} \end{array}\displaystyle \right ) . \end{aligned}$$

To yield improved search directions with superlinear convergence, our algorithm will solve two or three SLEs with the form of (10) with different perturbation vectors $(\zeta_{k},\mu^{k})$.

Subsequently, it is necessary to analyze the singularities of the coefficient matrix $V_{k}$ above, i.e., the solvability of SLE (10).

### Lemma 1

*For iterate* $x^{k}\in \tilde{X}_{0}$ *and* $z^{k}_{I_{k}}>0$, *if the matrix* $H_{k}$ *satisfies*

11 $$ H_{k}\succ \sum_{j\in I_{k}} \frac{z^{k}_{j}}{g_{j}^{k}}\nabla g_{j}^{k}\nabla g_{j}^{k^{T}}, $$

*then the coefficient matrix* $V_{k}$ *defined by* (9) *is invertible*, *where matrix order* $A\succ B$ *means* $(A-B)$ *is positive definite on* $R^{n}$.

### Proof

One knows that it is sufficient to show that SLE $V_{k}u=0$ has a unique solution zero, and this is elementary and omitted here. □

### Remark 2

Obviously, the positive definiteness request (11) on $H_{k}$ is weaker than the positive definiteness of $H_{k}$ itself on $R^{n}$. But it is stronger than the positive definiteness of $H_{k}$ on the null space of the gradients of approximate active constraints, i.e., on $\Omega_{k}:=\{d\in R^{n}: \nabla g_{I_{k}}(x ^{k})^{T}d=0\}$. However, the latter cannot ensure the invertibility of $V_{k}$.

Based on the above analysis and preparation, now we can describe the steps of our algorithm solving (P) as follows.

### Algorithm A

*Parameters:* $\alpha \in (0,\frac{1}{2}), \sigma,\beta, \theta, r\in (0,1), \xi \in (2,3)$, $\nu >2$, $\vartheta >1$, $M, p>0$; suitable small positive parameters $\gamma_{1}$, *γ* and $\gamma_{3}$; sufficiently small lower bound $\underline{\varepsilon}>0$ and sufficiently large upper bound $\overline{\varepsilon}>0$; termination accuracy $\epsilon >0$.

*Data:* $x^{0} \in \tilde{X_{0}}, \rho_{0}>0$, vectors $z^{0}$ with weights $z^{0}_{j}\in [\underline{\varepsilon}, \overline{\varepsilon}], j\in I$. Set $k:=0$.

**Step 1** *Compute working set*. Compute $\lambda^{k}$ by (5), $\Phi (x^{k},\lambda^{k})$ and $\delta (x^{k}, \lambda^{k})$ by (3)-(4). If $\Phi (x^{k},\lambda ^{k})\leq \epsilon $ or other suitable termination rule is satisfied, then $(x^{k},\lambda^{k})$ is an approximate KKT pair of problem (P) and stop; otherwise, generate the working sets $I^{\imath }_{k}$ and $I_{k}$ by (6).

**Step 2** *Yield matrix* $H_{k}$. Yield matrix $H_{k}$ such that it approximates to the Hessian of the Lagrangian associated with $(\mathrm{P}_{\rho_{k}})$ and satisfies request (11).

**Step 3** *Compute the main search directions*.

(i) Compute $(\bar{d}^{k},\bar{\lambda }^{k}_{I_{k}})$ by solving $\operatorname{SLE}(V_{k}; 1,0)$, see (10), then set $\bar{\lambda } ^{k}=(\bar{\lambda }^{k}_{I_{k}},0_{{I\setminus I_{k}}})=(\bar{ \lambda }^{k}_{\ell }, \bar{\lambda }^{k}_{\imath })$ with $\bar{ \lambda }^{k}_{\imath }=(\bar{\lambda }^{k}_{I^{\imath }_{k}}, 0_{ I ^{\imath }\setminus I^{\imath }_{k}})$.

(ii) Check conditions: (a) $\Vert \bar{d^{k}} \Vert \leq \gamma_{1}$, (b) $\bar{\lambda }^{k}\geq -\gamma_{2} e_{{I}}$, (c) $\bar{\lambda } ^{k}_{\ell }\ngtr\gamma_{3}e_{{I^{\ell }}}$. If all the three conditions above hold, then increase penalty parameter *ρ* by $\rho_{k+1}=\vartheta \rho_{{k}}$, set $x^{k+1}=x^{k}, z^{k+1}=z ^{k}, H_{k+1}=H_{k}$, $I^{\imath }_{k+1}=I^{\imath }_{k}, I_{k+1}=I _{k}$, $k:=k+1$, and go back to Step 3(i). Otherwise, set $\rho_{k+1}= \rho_{{k}}$, proceed to Step 3(iii) as follows.

(iii) Yield the weights of vector $\phi^{k}$ by

12 $$\begin{aligned} \phi_{j}^{k}=\min \bigl\{ 0,- \bigl( \operatorname{max} \bigl\{ -\bar{\lambda }_{j}^{k},0 \bigr\} \bigr)^{p}-Mg _{j}^{k} \bigr\} , \quad j\in I_{k}. \end{aligned}$$

Then compute

13 $$\begin{aligned}& \xi_{k}=\nabla f_{\rho_{k}} \bigl(x^{k} \bigr)^{T}\bar{d}^{k}-\sum_{j \in I_{k}} \frac{\bar{\lambda }_{j}^{k}\phi^{k}_{j}}{z^{k}_{j}}, \end{aligned}$$

14 $$\begin{aligned}& b_{k} = \bigl( \bigl\Vert \bar{d}^{k} \bigr\Vert ^{\nu }+ \bigl\Vert \phi^{k} \bigr\Vert \bigr) \biggl(\sum_{j\in I_{k}}\bar{ \lambda }^{k}_{j} \biggr)+\sum_{j\in I_{k}} \frac{\bar{\lambda }^{k} _{j}}{z^{k}_{j}} \phi^{k}_{j}, \end{aligned}$$

15 $$\begin{aligned}& \varphi_{k}= \textstyle\begin{cases} 1, &\mbox{if } b_{k}\leq 0; \\ \min\{\frac{(1-\theta )\vert \xi_{k} \vert }{b_{k}},1\}, &\mbox{if } b_{k}>0, \end{cases}\displaystyle \end{aligned}$$

and yield perturbation vectors via convex combinations

16 $$\begin{aligned} \mu^{k}=(1-\varphi_{k})\phi^{k}+ \varphi_{k} \bigl(- \bigl\Vert \bar{d}^{k} \bigr\Vert ^{\nu }- \bigl\Vert \phi^{k} \bigr\Vert \bigr)z^{k}_{I_{k}}. \end{aligned}$$

(iv) Compute $(d^{k},\lambda^{k}_{I_{k}})$ by solving $\operatorname{SLE}(V _{k}; 1,\mu^{k})$, see (10), then set $\lambda^{k}=(\lambda ^{k}_{I_{k}},0_{{I\setminus I_{k}}})=(\lambda^{k}_{\ell }, \lambda ^{k}_{\imath })$ with $\lambda^{k}_{\imath }=(\lambda^{k}_{I^{\imath }_{k}}, 0_{ I^{\imath }\setminus I^{\imath }_{k}})$.

**Step 4** *Trial of unit step.* If

$$ f_{\rho_{k}} \bigl(x^{k}+d^{k} \bigr)\leq f_{\rho_{k}} \bigl(x^{k} \bigr)+\alpha \nabla f_{ \rho_{k}} \bigl(x^{k} \bigr)^{T}d^{k}, \qquad g_{j} \bigl(x^{k}+d^{k} \bigr)< 0, \quad \forall j \in I, $$

then let the step size $t_{k}=1$, the high order correction direction $\tilde{d}^{k}=0$, and enter Step 7. Otherwise, proceed to Step 5.

**Step 5** *Generate high order correction direction.* Compute $(\tilde{d}^{k},\tilde{\lambda }^{k}_{I_{k}})$ by solving $\operatorname{SLE}(V _{k}; 0,\tilde{\mu }^{k})$, where

17 $$\begin{aligned}& \tilde{\mu }^{k} =-\omega_{k}e_{I_{k}}-Z_{k}g_{I_{k}} \bigl(x^{k}+d ^{k} \bigr), \end{aligned}$$

18 $$\begin{aligned}& \omega_{k}=\max \biggl\{ \bigl\Vert d^{k} \bigr\Vert ^{\xi }; \bigl\Vert d^{k} \bigr\Vert ^{2}\max \biggl\{ \biggl\vert 1-\frac{z_{j}^{k}}{\lambda_{j}^{k}} \biggr\vert ^{\sigma },j\in I_{k},\lambda^{k}_{j} \neq 0 \biggr\} \biggr\} . \end{aligned}$$

If $\Vert \tilde{d}^{k} \Vert >\Vert d^{k} \Vert $, reset $\tilde{d}^{k}=0$.

**Step 6** *Perform arc search.* Compute the step size $t_{k}$, the maximum number *t* of sequence $\{1,\beta,\beta^{2}, \ldots \}$ satisfying

19 $$\begin{aligned} & f_{\rho_{k}} \bigl(x^{k}+td^{k}+t^{2} \tilde{d}^{k} \bigr)\leq f_{\rho_{k}} \bigl(x ^{k} \bigr)+ \alpha t\nabla f_{\rho_{k}} \bigl(x^{k} \bigr)^{T}d^{k}, \end{aligned}$$

20 $$\begin{aligned} & g_{j} \bigl(x^{k}+td^{k}+t^{2} \tilde{d}^{k} \bigr)< 0,\quad j\in I. \end{aligned}$$

**Step 7** *Update.* Yield a new iterate by $x^{k+1}=x^{k}+t_{k}d^{k}+t^{{2}}_{k}\tilde{d}^{k}$ and compute

21 $$\begin{aligned} z^{k+1}_{j}=\min \bigl\{ \operatorname{max} \bigl\{ \bigl\Vert d^{k} \bigr\Vert ^{2}+\underline{ \varepsilon}, \lambda^{k}_{j} \bigr\} , \overline{\varepsilon} \bigr\} , \quad j \in I. \end{aligned}$$

Set $k:=k+1$, go back to Step 1.

Subsequently, we analyze and describe some properties of Algorithm A by the following lemma and several remarks. For convenience of writing, denote matrix

22 $$ Q_{k}:=H_{k}-\sum _{j\in I_{k}}\frac{z^{k}_{j}}{g_{j}^{k}} \nabla g_{j}^{k} \nabla g_{j}^{k^{T}}. $$

Then request (11) implies that matrix $Q_{k}$ is positive definite.

### Lemma 2

*For the directions* $\bar{d}^{k}$ *and* $d^{k}$ *yielded in Step* 3(i), (iv), *the following two relations hold*:

23 $$\begin{aligned}& \nabla f_{\rho_{k}} \bigl(x^{k} \bigr)^{T} \bar{d}^{k}=- \bigl(\bar{d}^{k} \bigr)^{T}Q_{k} \bar{d}^{k}\leq 0, \quad \forall k \geq 0, \end{aligned}$$

24 $$\begin{aligned}& \nabla f_{\rho_{k}} \bigl(x^{k} \bigr)^{T}d^{k} \leq \theta \xi_{k}\leq 0, \quad \forall k\geq 0. \end{aligned}$$

*Furthermore*, *when the iterative process goes into Step* 3(iii), (iv), *one has* $\bar{d}_{k}\neq0$ *and* $\xi_{k}<0$, *so* $d^{k}$ *is a feasible direction of descent of problem* $(\mathrm{P}_{\rho_{k}})$ *at point* $x^{k}$ *and the arc search in Step* 6 *can be finished by finite calculations*. *Therefore*, *Algorithm* A *is well defined*.

### Proof

First, from (9) and $\operatorname{SLE}(V _{k}; 1,0)$ (10), we have

$$\begin{aligned} \nabla f_{\rho_{k}} \bigl(x^{k} \bigr)^{T} \bar{d}^{k}&=- \bigl(\bar{d}^{k} \bigr)^{T} \biggl(H_{k} \bar{d}^{k}+\sum_{j\in I_{k}} \nabla g_{j}^{k}\bar{\lambda } ^{k}_{j} \biggr) \\ &=- \bigl(\bar{d}^{k} \bigr)^{T} \biggl(H_{k} - \sum_{j\in I_{k}}\frac{z ^{k}_{j}}{g_{j}^{k}}\nabla g_{j}^{k} \nabla g_{j}^{k^{T}} \biggr)\bar{d}^{k} \\ &=- \bigl(\bar{d}^{k} \bigr)^{T}Q_{k} \bar{d}^{k} \leq 0. \end{aligned}$$

So, conclusion (23) is at hand. Second, from (12)-(13), one gets

25 $$\begin{aligned} \phi^{k}_{j}\bar{\lambda }^{k}_{j}\geq 0, \quad \forall j\in I_{k}; \qquad \xi _{k} \leq \nabla f_{\rho_{k}} \bigl(x^{k} \bigr)^{T}\bar{d}^{k}\leq 0. \end{aligned}$$

On the other hand, taking into account $\operatorname{SLE}(V_{k}; 1,0)$ and $\operatorname{SLE}(V_{k}; 1,\mu^{k})$ as well as (13)-(14), it is not difficult to show that

26 $$\begin{aligned} \nabla f_{\rho_{k}} \bigl(x^{k} \bigr)^{T}d^{k}= \nabla f_{\rho_{k}} \bigl(x^{k} \bigr)^{T} \bar{d}^{k}-\sum_{j\in I_{k}} \frac{\bar{\lambda }^{k}_{j}\mu ^{k}_{j}}{z_{j}^{k}}= \xi_{k}+\varphi_{k}b_{k}. \end{aligned}$$

Again, in view of (15), it follows that $\varphi_{k}b_{k}=b _{k}\leq 0$ if $b_{k}\leq 0$, hence, the relations $\xi_{k}+\varphi _{k}b_{k}\leq \xi_{k}\leq \theta \xi_{k}$ hold since $\xi_{k}\leq 0$. If $b_{k}>0$, then $\xi_{k}+\varphi_{k}b_{k}\leq \xi_{k}+(\theta -1) \xi_{k} =\theta \xi_{k}$. In all, one gets $\xi_{k}+\varphi_{k}b_{k} \leq \theta \xi_{k}$. This, together with (26) and (25), shows that $\nabla f_{\rho_{k}}(x^{k})^{T}d^{k} \leq \theta \xi_{k}\leq 0$.

Third, if $\bar{d}^{k}=0$, then, from $\operatorname{SLE}(V_{k}; 1,0)$ (10), $g(x^{k})<0$ and (9), it follows that $\bar{\lambda }^{k}_{I_{k}}=0$. So, by the structure of Step 3, the iterate *k* does not go into Step 3(iii), (iv). Thus, $\bar{d}^{k} \neq0$ when the iterative process goes into Step 3(iii), (iv).

Finally, $\xi_{k}< 0$ follows from (25), (23) and $\bar{d}_{k}\neq0$. The remaining claims in Lemma 2 are at hand by $\xi_{k}<0$ and $g(x^{k})<0$. □

As an end of this section, to help the readers understand our algorithm, we further analyze the steps/structure of Algorithm A with three remarks below.

### Remark 3

Analysis for Step 3

(i) The role of solving $\operatorname{SLE}(V_{k}; 1,0)$ with no perturbation in Step 3(i) is to check whether the current iterate $x^{k}$ is an approximate KKT point of (P$_{\rho_{{k}}}$) and yield an ‘improved’ direction $\bar{d}^{k}$ to a certain extent.
(ii) If conditions (a) and (b) in Step 3(ii) are satisfied, and the parameters $\gamma_{1}$ and $\gamma_{2}$ are small enough, then $\operatorname{SLE}(V_{k}; 1,0)$ implies that $x^{k}$ is an approximate KKT point of (P$_{\rho_{{k}}}$). However, if case (c) is also satisfied, one cannot estimate $\Vert g_{\ell }(x^{k}) \Vert $. So, we increase the penalty parameter *ρ*. In practical computation, if conditions (a) and (b) are satisfied and $\Vert g_{\ell }(x^{k}) \Vert $ is small enough, we can terminate the algorithm.
(iii) From result (23), one knows that $\bar{d}^{k}$ is a descent direction of the merit function $f_{\rho_{k}}(x)$ at $x^{k}$ when $\bar{d}^{k}\neq0$. However, the primal feasibility and dual feasibility are relaxed to a large extent in $\operatorname{SLE}(V_{k}; 1,0)$, $\bar{d}^{k}$ cannot be used as an effective search direction. So, generally, the first direction $\bar{d}^{k}$ should be corrected by another SLE. For this goal, refer to [21], we construct and solve $\operatorname{SLE}(V_{k}; 1,\mu^{k})$ in Step 3(iii), (iv). Lemma 2 and the global convergence analysis in the next section show that the algorithm with search direction $d^{k}$ is well defined and globally convergent.

### Remark 4

Explanation for Steps 4 and 5

Usually, search direction $d^{k}$ cannot avoid the Maratos effect, i.e., unit step cannot be accepted by the associated line search for all sufficiently large iterates *k*. So, to overcome the Maratos effect and obtain superlinear convergence, one needs to compute an additional high order correction direction. Here, we generate it by solving $\operatorname{SLE}(V_{k}; 0,\tilde{\mu }^{k})$ in Step 5. Obviously, solving $\operatorname{SLE}(V_{k}; 0,\tilde{\mu }^{k})$ should add computational cost more or less. On the other hand, numerical testing shows that $d^{k}$ can still avoid the Maratos effect at some iterates. Therefore, to save computational cost as much as possible, the trial of unit step in Step 4 is added.

### Remark 5

With the help of the working set technique, the three SLEs solved in Algorithm A have a common coefficient matrix $V_{k}$, which can save the cost of computation and is different from those in Refs. [18, 26], etc. Furthermore, due to being interior point type and the constructing technique for $V_{k}$, Algorithm A is well defined at each iterate without any other CQ except the strict inner $\tilde{X}_{0}\neq\emptyset $, see Lemmas 1 and 2. In many existing QP-free type algorithms, see Refs. [1, 3, 21–24], the linearly independent constraint qualification (LICQ) is necessary to ensure the iterate itself is well defined. Of course, as we see in Assumption H3, to obtain the global and superlinear convergence of Algorithm A, a suitable CQ on the boundary of *X̃* is still necessary.

## Analysis of global convergence

In this section, we assume that the proposed algorithm (Algorithm A) generates an infinite iteration sequence $\{x^{k}\}$ of points. First, we show that the penalty parameter $\rho_{k}$ can be fixed after finite iterates. And then, we prove that Algorithm A is globally convergent. For this goal, the following hypotheses are necessary.

**H2**: Suppose that the sequences both $\{x^{k}\}$ and $\{H_{k}\}$ yielded by Algorithm A are bounded, and assume that there exists a positive constant *a* such that
  27 $$ d^{T}H_{k}d \geq a\Vert d \Vert ^{2}- \sum_{j\in I_{k}}\frac{z^{k}_{j}}{\vert g_{j}^{k} \vert } \bigl\Vert \nabla g_{j}^{k^{T}}d \bigr\Vert ^{2}, $$
  i.e., $d^{T}Q_{k}d\geq a\Vert d \Vert ^{2}, \forall k, \forall d \in R^{n}$.

**H3**: For each $x\in \tilde{X}$, suppose that
  (i) the gradient vectors $\{\nabla g_{j}(x), j\in I(x)\}$ are linearly independent; and
  (ii) if $x\notin X$, i.e., $g_{\ell }(x)\neq 0$, then there exist no scalars $\lambda_{j}\geq 0, j\in I(x)$ such that $\sum_{j\in I^{\ell }}\nabla g_{j}(x)=\sum_{j\in I(x)} \lambda_{j}\nabla g_{j}(x)$.

### Remark 6

Analysis for H2

The uniform ‘positive-definiteness’ request (27) on $\{H_{k}\}$ is weaker than the usual uniform positive-definiteness of $\{H_{k}\}$ itself on $R^{n}$, namely, $d^{T}H_{k}d\geq a\Vert d \Vert ^{2}, \forall k, \forall d\in R^{n}$. However, it is stronger than the uniform positive-definiteness of $H_{k}$ on the null space $\Omega_{k}$. It is encouraging that, based on the Lagrangian Hessian, we can design an alternative computational technique for $H_{k}$ such that $\{H_{k}\}$ is bounded and satisfies request (27), which implies (11) whenever $\{x^{k}\}$ is bounded, see formulas (52), (54) and (55) as well as Theorem 5 in Section 5.

### Remark 7

Analysis for H3

(i) Hypothesis H3 was introduced by Tits et al. in [1], Assumption 3. In our work, it plays two roles in the convergence analysis of Algorithm A. One is to ensure the correction for the penalty parameter *ρ* can be finished in a finite number of iterations, the other is to assure that the sequence $\{V_{k}\}$ of coefficient matrices is uniform invertible, see Lemmas 3 and 4. Furthermore, H3 is considerably milder than the linear independence of the gradients $\{\nabla g_{i}(x), i\in I^{ \ell }; \nabla g_{j}(x), j\in I^{\imath }(x)\}$, a detailed analysis for this assumption can be seen in [1, 32].
(ii) First, H3 automatically holds at each interior point $x\in \tilde{X}_{0}$. Second, H3 can be reduced to each accumulation point $x^{*}$ of the iterate sequence $\{x^{k}\}$, which satisfies $x^{*}\notin \tilde{X}_{0}$. However, the latter is difficult to be verified.

### Lemma 3

*Suppose that* H1, H2 *and* H3 *hold*. *Then the penalty parameter* $\rho_{k}$ *in Algorithm* A *is increased at most finite times*.

The proof of Lemma 3 is similar to the one of [1], Lemma 4.1, and omitted here. In what follows, *ρ̄* denotes the final value of $\rho_{k}$, i.e., $\rho_{k}\equiv \bar{\rho }$ when *k* is sufficiently large.

### Lemma 4

*Suppose that* H1, H2 *and* H3 *hold*. *Then*

(i) *the sequence* $\{V_{k}\}$ *of coefficient matrices is unified invertible*, *i*.*e*., *there exists a positive constant* *M̄* *such that* $\Vert V_{k}^{-1} \Vert \leq \bar{M}, \forall k\geq 0$, *and*
(ii) *both sequences* $\{(\bar{d}^{k},\bar{\lambda }^{k})\}$ *and* $\{(d^{k},\lambda^{k})\}$ *are bounded*.

### Proof

(i) By contradiction, suppose that there exists an infinite subset *K* such that $\Vert V_{k}^{-1} \Vert \stackrel{K}{\rightarrow }\infty $. In view of the boundedness of $\{x^{k}\}$ and $\{H_{k}\}$, Step 6 and the finite choice of $I^{\imath }_{k}$, without loss of generality, for $k\in K$, assume that

$$ I^{\imath }_{k}\equiv I',\qquad x^{k} \rightarrow x^{*}, \qquad H_{k}\rightarrow H_{*}, \qquad z^{k} \rightarrow z^{*}\geq \underline{ \varepsilon}e_{{I}}>0. $$

Denote $\hat{I}=I^{\ell }\cup I'$ and $Z_{*}=\operatorname{diag}(z^{*}_{ \hat{I}})$, then

28 $$\begin{aligned} V_{k}\stackrel{K}{\rightarrow } V_{*}:= \left ( \textstyle\begin{array}{c@{\quad}c@{}} H_{*} & \nabla g_{{\hat{I}}}(x^{*}) \\ Z_{*}\nabla g_{{\hat{I}}}(x^{*})^{T} & \operatorname{diag}(g_{{\hat{I}}}(x^{*})) \end{array}\displaystyle \right ) . \end{aligned}$$

Consequently, under H1-H3, refer to the proof of [21], Lemma 3.1(i), one can show that $V_{*}$ is nonsingular. So $\Vert V_{k}^{-1} \Vert \stackrel{K}{\rightarrow } \Vert V_{*}^{-1} \Vert <\infty $, which contradicts $\Vert V_{k}^{-1} \Vert \stackrel{K}{\rightarrow } \infty $.

(ii) First, the boundedness of $\{(\bar{d}^{k},\bar{\lambda }^{k})\}$ follows from $\operatorname{SLE}(V_{k}; 1,0)$ and conclusion (i) as well as $\rho_{k}\equiv \bar{\rho }$. Second, the boundedness of $\{\mu^{k}\}$ follows from formulas (12)-(16) and the boundedness of $\{(\bar{d}^{k},\bar{\lambda }^{k})\}$ as well as the positive boundary below of $\{z^{k}\}$. Therefore, the boundedness of $\{(d^{k},\lambda ^{k})\}$ is also at hand by $\operatorname{SLE}(V_{k}; 1,\mu^{k})$. □

### Lemma 5

*Suppose that* H1, H2 *and* H3 *hold*. *Let* $x^{*}$ *be an accumulation point of the sequence* $\{x^{k}\}$ *generated by Algorithm* A, *and suppose that* $\{x^{k}\}_{K}\rightarrow x^{*}$ *for some infinite index set K*. *If* $\{\xi_{k}\}_{K}\rightarrow 0$, *then* $x^{*}$ *is a KKT point of problem* $(\mathrm{P}_{\bar{\rho }})$, *and both* $\{\bar{\lambda }^{k}\}_{K}$ *and* $\{\lambda^{k}\}_{K}$ *converge to the unique multiplier vector* $\lambda^{*}$ *associated with* $x^{*}$.

### Proof

Let $(\bar{\lambda }^{*}; \hat{\lambda })$ be any given limit point of $\{(\bar{\lambda }^{k}; \lambda^{k})\}_{K}$. We first show that $(x^{*},\bar{\lambda }^{*})$ is a KKT pair of $(\mathrm{P}_{\bar{\rho }})$. In view of H2, Lemma 4 and the finite choice of $I^{\imath }_{k}$, we know that there is an infinite index $K'\subseteq K$ such that

29 $$ \begin{aligned} & I^{\imath }_{k}\equiv I', \qquad \bigl( \bar{ \lambda }^{k}; \lambda^{k} \bigr)\rightarrow \bigl(\bar{ \lambda }^{*} ; \hat{\lambda } \bigr), \\ &H_{k}\rightarrow H_{*},\qquad \bar{d} ^{k} \rightarrow \bar{d}^{*},\qquad z^{k}\rightarrow z^{*}\geq \underline{ \varepsilon} e_{I}, \quad k\in K'. \end{aligned} $$

Therefore, from (25), (23) and H2, one can easily get $\bar{d}^{*}=0$ by $\{\xi_{k}\}_{K}\rightarrow 0$. Further, taking the limit in $\operatorname{SLE}(V_{k}; 1,0)$ for $k\in K'$, we have, here $\hat{I}=I^{\ell }\cup I'$,

30 $$ \nabla f_{\bar{\rho }} \bigl(x^{*} \bigr)+\sum _{j\in \hat{I}}\bar{\lambda }^{*}_{j} \nabla g_{j} \bigl(x^{*} \bigr)=0; \qquad \bar{\lambda }_{j}^{*}g_{j} \bigl(x^{*} \bigr)=0, \quad \forall j\in \hat{I}. $$

Next, divert our attention to showing that $\bar{\lambda }^{*}\geq 0$. It is obvious that $\bar{\lambda }^{*}_{j}=0$ follows from $\bar{ \lambda }_{j}^{*}g_{j}(x^{*})=0$ for $j\in \hat{I}\setminus I(x^{*})$. Moreover, from the definition of $\xi_{k}$, i.e., (13), and $(\xi_{k},\bar{d}^{k})\stackrel{K'}{\rightarrow }(0,0)$, we can deduce that $\sum_{j\in \hat{I}}\frac{\bar{\lambda }^{k}_{j}\phi^{k}_{j}}{z ^{k}_{j}}\rightarrow 0, k\in K'$. Further, in view of (25), we know that each term $\frac{\bar{\lambda }^{k}_{j}\phi^{k}_{j}}{z ^{k}_{j}}\leq 0$, which together with (29) implies that $\bar{\lambda }^{k}_{j}\phi^{k}_{j}\stackrel{K'}{\rightarrow }0$. This, plus (12), shows that $\bar{\lambda }^{*}_{j}{\min}\{0,-( \operatorname{max}\{-\bar{\lambda }^{*}_{j},0\})^{p}-Mg_{j}(x^{*})\}=0$ for $j\in \hat{I}$, and this includes $\bar{\lambda }^{*}_{j}\geq 0$ for $j\in \hat{I}\cap I(x^{*})$. Therefore, $\bar{\lambda }^{*}_{\hat{I}} \geq 0$. Obviously, $\bar{\lambda }^{*}_{I\setminus \hat{I}}=0$. So $\bar{\lambda }^{*}\geq 0$ is at hand.

Hence, taking into account $x^{*}\in \tilde{X}$, we can conclude from (30) that $(x^{*},\bar{\lambda }^{*})$ is a KKT pair and $x^{*}$ is a KKT point for $(\mathrm{P}_{\bar{\rho }})$. Furthermore, the analysis above further shows that the sequence $\{\bar{\lambda }^{k} \}_{K}$ possesses a unique limit point, i.e., the unique KKT multiplier vector $\lambda^{*}$. So $\lim_{k\in K}\bar{\lambda }^{k}=\lambda^{*}$.

Finally, taking into account $(\bar{d}^{k},\bar{\lambda }^{k})\stackrel{K'}{ \rightarrow }(0,\lambda^{*})\geq 0$, from (12) and (16), we have $(\phi^{k}, \mu^{k})\stackrel{K'}{\rightarrow } 0$. Therefore, $\operatorname{SLE}(V_{k}; 1,\mu^{k})$ minus $\operatorname{SLE}(V_{k}; 1,0)$ gives

31 $$ V_{k}\left ( \textstyle\begin{array}{c} d^{k}-\bar{d}^{k} \\ \lambda^{k}_{\hat{I}}-\bar{\lambda }^{k}_{\hat{I}} \end{array}\displaystyle \right ) = \left ( \textstyle\begin{array}{c} 0 \\ \mu^{k} \end{array}\displaystyle \right ) \stackrel{K'}{\longrightarrow }\left ( \textstyle\begin{array}{c} 0 \\ 0 \end{array}\displaystyle \right ) . $$

This, along with Lemma 4(i), shows that $\hat{\lambda }= \lim_{k\in K'}\lambda^{k}=\lim_{k\in K'}\bar{\lambda } ^{k}=\lambda^{*}$. □

### Theorem 1

*Suppose that* H1, H2 *and* H3 *hold*. *Then each accumulation point* $x^{*}$ *of the sequence* $\{x^{k}\}$ *generated by Algorithm* A *is a KKT point of the original problem* (P), *i*.*e*. *problem* (1).

### Proof

First, there exists an infinite index set $K'$ such that $x^{k}\rightarrow x^{*}, k\in K'$, and relation (29) holds. By contradiction, suppose that $x^{*}$ is not a KKT point of (P). Then, from Lemma 4, without loss of generality, one can suppose that $\lambda^{k}=\bar{\lambda }^{k-1}-\bar{ \rho }\hat{e} \rightarrow \bar{\lambda }', k\in K'$. Therefore, it follows that $(x^{*},\bar{\lambda }')$ is not a KKT pair of (P), which further implies that $\delta (x^{*},\bar{\lambda }')>0$ and $I^{\imath }(x^{*})\subseteq I^{\imath }_{k}, k\in K'$ large enough. There are two cases as follows to be considered.

*Case I*: Assume that $x^{*}$ is a KKT point of $(\mathrm{P}_{\bar{\rho }})$. Then there exists a multiplier $\bar{\lambda }''\geq 0$ such that the KKT condition of $(\mathrm{P}_{\bar{\rho }})$ is satisfied at $(x^{*},\bar{\lambda }'')$. In view of $I^{\imath }(x^{*})\subseteq I ^{\imath }_{k}\equiv I'$ holds for $k\in K'$ large enough, it is easy to know, from the KKT condition of $(\mathrm{P}_{\bar{\rho }})$, that $(0,\bar{\lambda }''_{I^{*}})$ is a solution to SLE in $(u,v)$

32 $$\begin{aligned} V_{*}\left ( \textstyle\begin{array}{c} u \\ v \end{array}\displaystyle \right ) =\left ( \textstyle\begin{array}{c} -\nabla f_{\bar{\rho }}(x^{*}) \\ 0 \end{array}\displaystyle \right ), \end{aligned}$$

where matrix $V_{*}$ is defined by (28). On the other hand, passing to the limit in $\operatorname{SLE}(V_{k}; 1,0)$ for $k\in K'$ and $k\rightarrow \infty $, one knows that $(\bar{d}^{*},\bar{\lambda } ^{*}_{I^{*}})$ also solves system (32) above. Taking into account the nonsingularity of matrix $V_{*}$ (by Lemma 4(i)), one knows that the solution of (32) is unique. So $\bar{d}^{*}=0$ and $\bar{\lambda }^{*}_{I^{*}}=\bar{ \lambda }''_{I^{*}}\geq 0$, which implies $\bar{\lambda }^{*}=\bar{ \lambda }''\geq 0$. Thus, conditions (a) and (b) in Step 3(ii) are always satisfied for $k\in K'$ large enough. Therefore, in view of $\rho_{k}\equiv \bar{\rho }<\infty $ for *k* large enough, Step 3(ii) implies $\bar{\lambda }^{k}_{{I^{\ell }}}>\gamma_{3} e_{{I^{\ell }}} $ for $k\in K'$ large enough, which further implies that $\bar{\lambda }^{*}_{{I^{\ell }}}\geq \gamma_{3} e_{{I^{\ell }}}>0$. Hence, it follows from the complementary slackness at KKT pair $(x^{*},\bar{ \lambda }'')$, $0=\bar{\lambda }''_{j}g_{j}(x^{*})=\bar{\lambda }^{*} _{j}g_{j}(x^{*})=0$ ($j\in I^{\ell }$). So $g_{\ell }(x^{*})=0$, which together with Proposition 1 implies that $x^{*}$ is also a KKT point of (P), which contradicts the assumption that $x^{*}$ is not a KKT point of (P).

*Case II*: Suppose that $x^{*}$ is not a KKT point of ($\mathrm{P}_{\bar{\rho }}$). And, by Lemma 5 and $\xi_{k}\leq 0$, one can deduce that $\xi_{k}\rightarrow \bar{\xi }<0, k\in K'$. Further, this along with (13) and (23) as well as H2, shows that $\lim_{k\in K'}(\Vert \bar{d}^{k} \Vert ^{\nu }+\Vert \phi^{k} \Vert )>0$. So there exist a subset $K''\subseteq K'$ and a positive constant *ϖ* such that

$$\begin{aligned} \xi_{k}\leq \bar{\xi }/2 < 0,\qquad \bigl( \bigl\Vert \bar{d}^{k} \bigr\Vert ^{\nu }+ \bigl\Vert \phi^{k} \bigr\Vert \bigr) \geq \varpi > 0,\quad k\in K''. \end{aligned}$$

The remaining proof is divided into two steps.

*Step A:* Show that there exists a constant $\bar{t}>0$ such that the step-length $t_{k}\geq \bar{t}$ holds for all $k\in K''$.

(A1) Analyze inequality (20). First, for $j\notin I(x ^{*}), g_{j}(x^{*})<0$, from the boundedness of $\{(d^{k},\tilde{d} ^{k})\}_{K''}$ and the continuity of $g_{j}$, one gets that $g_{j}(x^{k}+td^{k}+t^{2}\tilde{d}^{k})<0$ holds for $k\in K''$ large enough and $t>0$ sufficiently small. Second, consider index $j\in I(x^{*})$, i.e., $g_{j}(x^{*})=0$. In view of $I^{\imath }(x ^{*})\subseteq I^{\imath }_{k}$, which implies $j\in I_{k}$, from Taylor expansion, formulas (9), (16) and $\operatorname{SLE}(V_{k}; 1,\mu^{k})$ as well as $\Vert \tilde{d}^{k} \Vert \leq \Vert \bar{d}^{k} \Vert $, for $t>0$ small enough, we obtain that

$$\begin{aligned} g_{j} \bigl(x^{k}+td^{k}+t^{2} \tilde{d}^{k} \bigr)&=g_{j}^{k}+t\nabla g_{j}^{k ^{T}}d^{k}+ o(t) =g_{j}^{k}+t \frac{\mu_{j}^{k}-\lambda^{k}_{j}g_{j} ^{k}}{z^{k}_{j}}+o(t) \\ &= \biggl(1-t\frac{\lambda^{k}_{j}}{z^{k}_{j}} \biggr)g_{j}^{k}+t \frac{1-\varphi _{k}}{z^{k}_{j}}\phi_{j}^{k} -t\varphi_{k} \bigl( \bigl\Vert \bar{d}^{k} \bigr\Vert ^{\nu }+ \bigl\Vert \phi^{k} \bigr\Vert \bigr)+o(t) \\ &\leq -t\varphi_{k} \bigl( \bigl\Vert \bar{d}^{k} \bigr\Vert ^{\nu }+ \bigl\Vert \phi^{k} \bigr\Vert \bigr)+o(t), \end{aligned}$$

where the last inequality follows from Lemma 4(ii), $z^{k}_{j}\geq \underline{\varepsilon}$, $\varphi_{k}\leq 1$ and $\phi^{k}_{j}\leq 0$.

On the other hand, taking into account $\xi_{k}\leq \bar{\xi }/2<0$ and the boundedness of $b_{k}$ (14) (by Lemma 4) as well as (15), we know that there exists a constant $\varphi >0$ such that $\varphi_{k}\geq \varphi >0, k\in K''$. So $g_{j}(x^{k}+td^{k}+t ^{2}\tilde{d}^{k})\leq -\varphi \varpi t+o(t)<0$ holds for $k\in K''$ large enough and $t>0$ sufficiently small. Therefore, inequality (20) holds for $t>0$ sufficiently small and $k\in K''$ large enough.

(A2): Analyze inequality (19). From Taylor expansion and (24), one gets
  $$\begin{aligned} f_{\bar{\rho }} \bigl(x^{k}+td^{k}+t^{2} \tilde{d}^{k} \bigr)-f_{\bar{\rho }} \bigl(x ^{k} \bigr)- \alpha t\nabla f_{\bar{\rho }} \bigl(x^{k} \bigr)^{T}d^{k} & =(1-\alpha )t \nabla f_{\bar{\rho }} \bigl(x^{k} \bigr)^{T}d^{k}+o(t) \\ & \leq (1-\alpha )t\theta \xi_{k}+o(t) \\ & \leq (1-\alpha )t\theta \bar{\xi }/2+o(t) \\ & \leq 0. \end{aligned}$$

Hence, inequality (19) holds for $k\in K''$ large enough and $t>0$ sufficiently small. Up to now, one can conclude that there exists a constant $\bar{t}>0$ such that $t_{k}\geq \bar{t}$ for each $k\in K''$.

*Step B:* Use $t_{k}\geq \bar{t}>0$ ($k\in K''$) to bring a contradiction. Because of $\lim_{k\in K''}f_{\bar{\rho }}(x ^{k})=f_{\bar{\rho }}(x^{*})$ and the monotone property of $\{f_{\bar{ \rho }}(x^{k})\}$, one knows that $\lim_{k\rightarrow \infty }f _{\bar{\rho }}(x^{k})=f_{\bar{\rho }}(x^{*})$. Further, in view of (19) and (24), it follows that for $k\in K''$ large enough

$$\begin{aligned} f_{\bar{\rho }} \bigl(x^{k+1} \bigr)-f_{\bar{\rho }} \bigl(x^{k} \bigr)\leq \alpha t_{k} \nabla f_{\bar{\rho }} \bigl(x^{k} \bigr)^{T}d^{k}\leq \alpha t_{k}\theta \xi_{k} \leq \alpha \theta \bar{\xi }\bar{t}/2. \end{aligned}$$

Passing to the limit for $k\in K''$ and $k\rightarrow \infty $ in the inequality above, we can bring a contradiction. Summarizing the discussions above, the whole proof of Theorem 1 is completed. □

## Analysis of strong and superlinear convergence

In this part, under some additional mild assumptions, we first show that the proposed algorithm is strongly convergent, that is, the whole sequence $\{x^{k}\}$ is convergent. Then the unit step can be accepted and the Maratos effect can be avoided for all *k* large enough. At last, we prove that Algorithm A achieves superlinear convergence.

**H4**
  (i) The functions $f(x)$ and $g(x)$ are all twice continuously differentiable over *X̃*; and
  (ii) there exists an accumulation point $x^{*}$ of the sequence $\{x^{k}\}$ of iterative points with (unique) KKT multiplier $\lambda '$ associated with (P) such that the second order sufficiency conditions (SOSC) and the strict complementarity hold, i.e., the KKT pair $(x^{*}, \lambda ')$ of (P) satisfies $\lambda '_{{I^{\imath }(x^{*})}}>0$ and
    $$\begin{aligned} d^{T}\nabla_{xx}^{2} L \bigl(x^{*}, \lambda ' \bigr)d>0,\quad \forall d\in \bigl\{ d\in R^{n}: d \neq 0, \nabla g_{{I(x^{*})}} \bigl(x^{*} \bigr)^{T}d=0 \bigr\} . \end{aligned}$$

### Remark 8

Denote the Lagrangian function of problem $(\mathrm{P}_{\bar{\rho }})$ by $L_{\bar{\rho }}(x,\lambda )=f_{\bar{ \rho }}(x)+\sum_{j\in I}\lambda_{j} g_{j}(x)$. Then, with relation $\lambda_{\bar{\rho }}=\lambda -\bar{\rho }\hat{e}$, we have $L(x,\lambda_{\bar{\rho }})=L_{\bar{\rho }}(x,\lambda )$. Therefore, taking into account Lemma 6(iv), it is readily checked that the SOSC with the strict complementarity for $(\mathrm{P}_{\bar{\rho }})$ is identical with that for (P).

### Lemma 6

*Suppose that* $\tilde{X}\neq\emptyset $ *and assumptions* H2, H3 *and* H4 *are satisfied* (*by Remark* 1, $\tilde{X}\neq\emptyset$ *plus* H3(i) *implies* $\tilde{X}_{0}\neq\emptyset$). *Then*, *for any subset* *K* *such that* $\{x^{k}\}_{K}$ *converges to the limit point* $x^{*}$ *stated in* H4, *there exists an infinite subset* $K'\subseteq K$ *such that*

(i) $I^{\imath }(x^{*})\subseteq I^{\imath }_{k}$ *for* $k\in K'$ *sufficiently large*;
(ii) $x^{*}$ *is a KKT point of problem* $(\mathrm{P}_{\bar{\rho}})$;
(iii) $\{(\bar{d}^{k},\bar{\lambda }^{k})\}_{K'}\rightarrow (0, \lambda^{*})$ *and* $\{(d^{k},\lambda^{k})\}_{K'}\rightarrow (0,\lambda ^{*})$, *where* $\lambda^{*}$ *together with* $x^{*}$ *is a KKT pair of problem* $(\mathrm{P}_{\bar{\rho }})$; *and*
(iv) *the KKT multiplier* $\lambda '$ *of* (P) *and* $\lambda ^{*}$ *of* $(\mathrm{P}_{\bar{\rho }})$ *associated with the KKT point* $x^{*}$ *satisfy* $\lambda '=\lambda^{*}-\bar{\rho }\hat{e}, \lambda ^{*}_{{I(x^{*})}}>0$.

### Proof

(i) From Lemma 4(ii), there exists an infinite subset $K'\subseteq K$ such that

$$\begin{aligned} x^{k}\rightarrow x^{*}, \qquad \lambda^{k}= \bigl(\bar{ \lambda }^{k-1}-\bar{ \rho }\hat{e} \bigr)\rightarrow \bar{ \lambda }',\quad k\in K'. \end{aligned}$$

If $(x^{*},\bar{\lambda }')$ is a KKT pair of (P), then $\bar{\lambda }'=\bar{\lambda }^{*}$. Further, under H4, by [24, 29], one knows that $I^{\imath }_{k}\equiv I^{\imath }(x^{*})$ for $k\in K'$ large enough. Otherwise, we have $0<\delta (x^{*},\bar{\lambda }')\stackrel{K'}{ \leftarrow }\delta (x^{k}, \lambda^{k})$. So, from (6), $I^{\imath }(x^{*})\subseteq I^{\imath }_{k}$ also holds for $k\in K'$ large enough.

(ii) By contradiction, suppose that $x^{*}$ is not a KKT point of $(\mathrm{P}_{\bar{\rho }})$. Then, taking into account conclusion $I^{\imath }(x^{*})\subseteq I^{\imath }_{k}$ ($k\in K'$ large enough), by Case II of the proof of Theorem 1, we can bring a contradiction.

(iii) To show $\{(\bar{d}^{k},\bar{\lambda }^{k})\}_{K'}\rightarrow (0, \lambda^{*})$, it is sufficient to show that $(0,\lambda^{*})$ is a unique accumulation point of $\{(\bar{d}^{k},\bar{\lambda }^{k})\} _{K'}$. Let $(\bar{d},\bar{\lambda })$ be any given accumulation point of $\{(\bar{d}^{k},\bar{\lambda }^{k})\}_{K'}$. Since the sequences $\{(\bar{d}^{k},\bar{\lambda }^{k})\}$ and $\{z^{k}\}$ are all bounded, in view of H2, H3 and $I^{\imath }_{k}\subseteq I^{\imath }$, there exists an infinite subset $K''\subseteq K'$ such that

33 $$\begin{aligned} I^{\imath }_{k}\equiv I',\qquad H_{k}\rightarrow H_{*}, \qquad \bigl(\bar{d}^{k}, \bar{ \lambda }^{k} \bigr)\rightarrow (\bar{d},\bar{\lambda }),\qquad z^{k} \rightarrow z^{*},\quad k\in K''. \end{aligned}$$

Now, passing to the limit for $k\in K''$ and $k\rightarrow \infty $ in $\operatorname{SLE}(V_{k}; 1,0)$, we deduce that ($\bar{d},\bar{\lambda }_{\hat{I}}$) ($\hat{I}:=I^{\ell }\cup I'$) solves SLE (32). Further, it follows from Lemma 4(i) that the coefficient matrix of SLE (32) is nonsingular. Thus the solution of (32) is unique. On the other hand, in view of $I^{\imath }(x^{*})\subseteq I', I^{\ell }(x^{*})=I^{\ell }$ and $(x^{*},\lambda ^{*})$ being a KKT pair of $(\mathrm{P}_{\bar{\rho }})$, we know that $(0,\lambda^{*}_{\hat{I}})$ is also a solution to system (32). Therefore $(\bar{d},\bar{\lambda }_{\hat{I}})=(0, \lambda^{*}_{\hat{I}})$, this further implies that $(\bar{d},\bar{ \lambda })=(0,\lambda^{*})$ and $(0,\lambda^{*})$ is a unique limit point of $\{(\bar{d}^{k},\bar{\lambda }^{k})\}_{K'}$.

Finally, conclusion $\{(d^{k},\lambda^{k})\}_{K'}\rightarrow (0,\lambda ^{*})$ follows from $\{(\bar{d}^{k},\bar{\lambda }^{k})\}_{K'} \rightarrow (0,\lambda^{*})$ and (31).

(iv) By Proposition 1 and $g_{\ell }(x^{*})=0$, we have $\lambda '=\lambda^{*}-\bar{\rho }\hat{e}$, and $\lambda^{*}_{{I^{ \imath }(x^{*})}}=\lambda '_{{I^{\imath }(x^{*})}}>0$ by H4(ii). Further, in view of $\bar{d}^{k}\rightarrow 0, \bar{\lambda }^{k} \rightarrow \lambda^{*}\geq 0, k\in K'$, one knows that conditions (a) and (b) in Step 3(ii) hold for *k* large enough. Therefore, taking into account $\rho_{k}\equiv \bar{\rho }$ for *k* large enough, it follows that $\bar{\lambda }^{k}> \gamma e_{{I^{\ell }}}$ by Step 3(ii), so $\lambda^{*}_{\ell }\geq \gamma_{3} e_{{I^{\ell }}}>0$. Therefore $\lambda^{*}_{{I(x^{*})}}>0$ holds. □

### Remark 9

In view of $I^{\ell }(x^{*})=I^{\ell }$, from H3, H4 and Lemma 6(ii), (iv), the following conclusion holds: The LICQ, SOSC and strict complementarity of problem (P) and problem (P$_{\bar{\rho }}$) are satisfied at their KKT pair $(x^{*},\lambda ')$ and $(x^{*},\lambda^{*})$, respectively.

In view of Remark 9, similarly to the proof of [21], Theorem 4.1, Lemma 4.2, we have the following result.

### Theorem 2

*Suppose that* $\tilde{X}\neq\emptyset $ *and assumptions* H2, H3 *and* H4 *are satisfied*. *Then*

(i) $x^{k}\rightarrow x^{*}$, *i*.*e*., *Algorithm* A *is strongly convergent*;
(ii) $(\bar{d}^{k},\bar{\lambda }^{k})\rightarrow (0,\lambda ^{*}), (d^{k},\lambda^{k})\rightarrow (0,\lambda^{*}), z^{k}\rightarrow \min \{\max \{\underline{\varepsilon} e_{I},\lambda^{*}\},\overline{ \varepsilon}e_{I}\}$, *and*
(iii) $\phi^{k}=0, \mu^{k}=-\varphi_{k}\Vert \bar{d}^{k} \Vert ^{ \nu }z^{k}_{I_{k}}$, $I^{\imath }_{k}\equiv I^{\imath }(x^{*})$ *and* $I_{k}\equiv I_{*}:=I(x^{*})$ *if* *k* *is sufficiently large*.

### Lemma 7

*Suppose that the hypotheses in Lemma* 6 *hold*, *and assume that the boundary parameters* $\underline{\varepsilon}$ *and* *ε̅* *satisfy*

34 $$\begin{aligned} \underline{\varepsilon}\leq \min \bigl\{ \lambda^{*}_{j},j \in I_{*} \bigr\} , \qquad \overline{\varepsilon}\geq \max \bigl\{ \lambda^{*}_{j},j\in I_{*} \bigr\} . \end{aligned}$$

*Then*

35 $$\begin{aligned} z^{k}_{I_{*}}\rightarrow \lambda^{*}_{I_{*}}, \qquad \omega_{k}=o \bigl( \bigl\Vert d^{k} \bigr\Vert ^{2} \bigr), \end{aligned}$$

*and the solution* $(\tilde{d}^{k},\tilde{\lambda }^{k}_{I_{*}})$ *of* $\operatorname{SLE}(V_{k};0,\tilde{\mu }^{k}) $ *satisfies*

36 $$ \Vert \bigl(\tilde{d}^{k}, \tilde{ \lambda}^{k}_{I_{*}} \bigr) \Vert =O(\hat{\omega}_{k})= o \bigl( \bigl\Vert d^{k} \bigr\Vert \bigr), \qquad \hat{ \omega}_{k} \bigl\Vert d^{k} \bigr\Vert =o( \omega_{k}), $$

*where*

37 $$ \hat{\omega }_{k}=\max \bigl\{ \bigl\vert z^{k}_{j}/\lambda^{k}_{j}-1 \bigr\vert \cdot \bigl\Vert d ^{k} \bigr\Vert , j\in I_{*}; \bigl\Vert d^{k} \bigr\Vert ^{2} \bigr\} . $$

*Furthermore*, *the correction direction* $\tilde{d}^{k}$ *in Step* 6 *is always yielded by the solution of* $\operatorname{SLE}(V_{k};0,\tilde{\mu } ^{k})$.

### Proof

First, from the given conditions and Theorem 2(iv), relation $z^{k}_{I_{*}}\rightarrow \lambda^{*} _{I_{*}}$ is at hand. Further, this, together with Theorem 2(ii), shows that $z^{k}_{j}/\lambda^{k}_{j}\rightarrow 1$ for $j\in I_{*}$. So, it follows that $\omega_{k}=o(\Vert d^{k} \Vert ^{2})$ from (18).

Second, we prove relation (36). From Theorem 2(ii), (iii), we know that $\mu^{k}=-\varphi_{k}\Vert \bar{d}^{k} \Vert ^{\nu } z^{k}_{I_{*}}\rightarrow 0, k\rightarrow \infty $. This, along with $\operatorname{SLE}(V_{k};1,0)$ and $\operatorname{SLE}(V_{k};1,\mu^{k})$ as well as Lemma 4(i), implies that there exists a positive constant *c* such that

38 $$ \bigl\Vert d^{k}-\bar{d}^{k} \bigr\Vert \leq c \bigl\Vert \bar{d}^{k} \bigr\Vert ^{\nu }, \qquad \bigl\Vert \lambda^{k}-\bar{ \lambda}^{k} \bigr\Vert \leq c \bigl\Vert \bar{d}^{k} \bigr\Vert ^{\nu }, \qquad \bigl\Vert d^{k} \bigr\Vert \sim \bigl\Vert \bar{d} ^{k} \bigr\Vert . $$

Therefore, from definition (17) of $\tilde{\mu }^{k}$, Taylor expansion and $\operatorname{SLE}(V_{k};1,\mu^{k})$, one has for $j\in I_{*}$

$$\begin{aligned} \tilde{\mu }^{k}_{j}&=-\omega_{k}-z^{k}_{j}g_{j} \bigl(x^{k}+d^{k} \bigr) \\ &=-\omega_{k}-z^{k}_{j} \bigl(g_{j}^{k}+ \nabla g_{j}^{k^{T}}d^{k} \bigr)+O \bigl( \bigl\Vert d^{k} \bigr\Vert ^{2} \bigr) \\ &=-\omega_{k}-z^{k}_{j} \bigl(1-z_{j}^{k}/ \lambda^{k}_{j} \bigr)\nabla g_{j}^{k ^{T}}d^{k}+O \bigl( \bigl\Vert d^{k} \bigr\Vert ^{2} \bigr) \\ &=-\omega_{k}+O \bigl(\max \bigl\{ \bigl\vert z^{k}_{i}/ \lambda^{k}_{i}-1 \bigr\vert \cdot \bigl\Vert d^{k} \bigr\Vert , i\in I_{*} \bigr\} \bigr)+O \bigl( \bigl\Vert d^{k} \bigr\Vert ^{2} \bigr) \\ &=-\omega_{k}+O(\hat{\omega }_{k})+O \bigl( \bigl\Vert d^{k} \bigr\Vert ^{2} \bigr) \\ &=o \bigl( \bigl\Vert d^{k} \bigr\Vert ^{2} \bigr)+O( \hat{\omega}_{k})+O \bigl( \bigl\Vert d^{k} \bigr\Vert ^{2} \bigr). \end{aligned}$$

Obviously, definition (37) implies $\Vert d^{k} \Vert ^{2}=O( \hat{\omega }_{k})$. Thus $\Vert \tilde{\mu }^{k} \Vert =O(\hat{\omega }_{k})=o( \Vert d^{k} \Vert )$. Therefore, from $\operatorname{SLE}(V_{k};0,\tilde{\mu }^{k})$ and Lemma 4, it is clear that the first relation of (36) holds. Finally, relation $\hat{\omega }_{k}\Vert d^{k} \Vert =o( \omega_{k})$ follows from definitions (37) of $\hat{\omega } _{k}$ and (18) of $\omega_{k}$. □

To ensure the step size $t_{k}\equiv 1$ for *k* large enough, which is necessary to obtain superlinear convergence, similarly or refer to [8, 9], the following second order approximate condition is necessary.

**H5**: Assume that the relation $\Vert P_{k}(\nabla_{xx}^{2}L_{\bar{\rho }}(x^{k},\lambda^{k})-H_{k})P_{k}\bar{d}^{k} \Vert =o( \Vert \bar{d}^{k} \Vert )$ holds, where the projective matrix $P_{k}$ is defined by $P_{k}=E_{n}-N_{k}(N_{k}^{T}N_{k})^{-1}N_{k}^{T}$ with $N_{k}= \nabla g_{{I_{*}}}(x^{k})$ and *n*-order unit matrix $E_{n}$.

### Remark 10

About H5

(i) Due to $I_{*}=I(x^{*})=I ^{\ell }\cup I^{\imath }(x^{*})$, one knows from H3(i) that matrix $N_{k}\rightarrow \nabla g_{{I_{*}}}(x^{*})$ which is column full rank, and matrix $P_{k}$ is well defined when *k* is large enough.
(ii) The 2-sided projection second order approximation H5 above, also used in [1, 8, 18, 22], is milder than the 1-sided projection second order approximation:
  $\mathbf{H5^{+}}$
$\Vert P_{k}(\nabla_{xx}^{2}L_{\bar{\rho }}(x^{k},\lambda^{k})-H_{k})\bar{d}^{k} \Vert =o( \Vert \bar{d}^{k} \Vert )$.
  Both the two can ensure the step unit is achieved. However, the associated algorithms can attain (one-step) q-superlinear convergence under the latter, and only two-step superlinear convergence under the former.
(iii) In view of relation (38), assumptions H5 and H5^+^ are equivalent to $\Vert P_{k}(\nabla_{xx}^{2}L_{\bar{\rho }}(x^{k},\lambda^{k})-H_{k})P_{k}d^{k} \Vert =o( \Vert d^{k} \Vert )$ and $\Vert P_{k}(\nabla_{xx}^{2}L_{\bar{\rho }}(x^{k},\lambda^{k})-H_{k})d^{k} \Vert =o( \Vert d^{k} \Vert )$, respectively.

### Theorem 3

*Suppose that* $\tilde{X}\neq\emptyset $ *and hypotheses* H2-H5 *hold*, *and assume that the boundary parameters* $\underline{\varepsilon}$ *and* *ε̅* *satisfy* (34). *Then the step size* $t_{k}$ *of Algorithm* A *always equals one*, *i*.*e*., $t_{k} \equiv 1$ *for* *k* *large enough*.

### Proof

(i) Discuss (20). For $j\notin I_{*}=I(x ^{*})$, $g_{j}(x^{*})<0$, using the continuity of $g_{j}$ and $(x^{k},d^{k},\tilde{d}^{k})\rightarrow (x^{*},0,0), k\rightarrow \infty $, we know that (20) holds for $t=1$ and *k* large enough.

For $j\in I_{*}=I(x^{*})=I_{k}$, in view of $\operatorname{SLE}(V_{k};1,\mu ^{k})$, Theorem 2(iii) and $\Vert d^{k} \Vert \sim \Vert \bar{d}^{k} \Vert $ as well as $\lambda^{k}_{j}\rightarrow \lambda^{*}_{j}>0$, we have

39 $$\begin{aligned} z^{k}_{j}\nabla g_{j}^{k^{T}}d^{k}+g_{j}^{k} \lambda^{k}_{j}=\mu^{k} _{j}=o \bigl( \bigl\Vert d^{k} \bigr\Vert ^{2} \bigr),\quad g_{j}^{k}=O \bigl( \bigl\Vert d^{k} \bigr\Vert \bigr). \end{aligned}$$

Again, taking into account $\operatorname{SLE}(V_{k};0,\tilde{\mu }^{k})$, one has

$$\begin{aligned} z^{k}_{j}\nabla g_{j}^{k^{T}} \tilde{d}^{k}+z^{k}_{j}g_{j} \bigl(x^{k}+d^{k} \bigr)+ \tilde{\lambda }^{k}_{j}g_{j}^{k}=- \omega_{k}. \end{aligned}$$

This, together with (39), (36) and $\omega_{k}=o(\Vert d^{k} \Vert ^{2})$, shows that

40 $$\begin{aligned} g_{j} \bigl(x^{k}+d^{k} \bigr)+\nabla g_{j}^{k^{T}}\tilde{d}^{k}&=-\frac{\omega _{k}}{z^{k}_{j}}+ \tilde{\lambda }^{k}_{j}O \bigl( \bigl\Vert d^{k} \bigr\Vert \bigr) =- \frac{\omega _{k}}{z^{k}_{j}}+O \bigl(\hat{\omega }_{k} \bigl\Vert d^{k} \bigr\Vert \bigr) \\ &=-\frac{\omega_{k}}{z^{k}_{j}}+o(\omega_{k}) =O(\omega_{k})=o \bigl( \bigl\Vert d ^{k} \bigr\Vert ^{2} \bigr). \end{aligned}$$

Further, using Taylor expansion and (36), one has

$$\begin{aligned} g_{j} \bigl(x^{k}+d^{k}+\tilde{d}^{k} \bigr)&=g_{j} \bigl(x^{k}+d^{k} \bigr)+\nabla g_{j} \bigl(x ^{k}+d^{k} \bigr)^{T} \tilde{d}^{k}+O \bigl( \bigl\Vert \tilde{d}^{k} \bigr\Vert ^{2} \bigr) \\ &=g_{j} \bigl(x^{k}+d^{k} \bigr)+\nabla g_{j}^{k^{T}}\tilde{d}^{k} +O \bigl( \bigl\Vert d^{k} \bigr\Vert \cdot \bigl\Vert \tilde{d}^{k} \bigr\Vert \bigr)+O \bigl( \bigl\Vert \tilde{d}^{k} \bigr\Vert ^{2} \bigr) \\ &=-\frac{\omega_{k}}{z^{k}_{j}}+o(\omega_{k})+O \bigl(\hat{\omega }_{k} \bigl\Vert d ^{k} \bigr\Vert \bigr) =- \frac{\omega_{k}}{z^{k}_{j}}+o(\omega_{k}) =o \bigl( \bigl\Vert d^{k} \bigr\Vert ^{2} \bigr). \end{aligned}$$

Hence, we can conclude from the fourth equality above that inequality (20) holds for $j\in I_{*}$, $t=1$ and *k* large enough since $z^{k}_{j}\rightarrow \lambda^{*}_{j}>0$.

(ii) Analyze (19). From Taylor expansion and (36), it follows that

41 $$\begin{aligned} w_{k}&:= f_{\bar{\rho }} \bigl(x^{k}+d^{k}+ \tilde{d}^{k} \bigr)-f_{\bar{\rho }} \bigl(x ^{k} \bigr)- \alpha \nabla f_{\bar{\rho }} \bigl(x^{k} \bigr)^{T}d^{k} \\ &=\nabla f_{\bar{\rho }} \bigl(x^{k} \bigr)^{T} \bigl(d^{k}+\tilde{d}^{k} \bigr)+\frac{1}{2} \bigl(d ^{k} \bigr)^{T} \nabla^{2}f_{\bar{\rho }} \bigl(x^{k} \bigr)d^{k}-\alpha \nabla f_{\bar{ \rho }} \bigl(x^{k} \bigr)^{T}d^{k}+o \bigl( \bigl\Vert d^{k} \bigr\Vert ^{2} \bigr). \end{aligned}$$

On the other hand, from $\operatorname{SLE}(V_{k};1,\mu^{k})$, we have

42 $$\begin{aligned} H_{k}d^{k}+\nabla f_{\bar{\rho }} \bigl(x^{k} \bigr)+\sum_{j\in I_{*}} \lambda^{k}_{j}\nabla g_{j}^{k}=0, \end{aligned}$$

which, together with (36), gives

43 $$\begin{aligned} &\nabla f_{\bar{\rho }} \bigl(x^{k} \bigr)^{T}d^{k}=- \bigl(d^{k} \bigr)^{T}H_{k}d^{k}- \sum_{j\in I_{*}}\lambda^{k}_{j}\nabla g_{j}^{k^{T}}d^{k}, \end{aligned}$$

44 $$\begin{aligned} &\nabla f_{\bar{\rho }} \bigl(x^{k} \bigr)^{T} \bigl(d^{k}+\tilde{d}^{k} \bigr)=- \bigl(d^{k} \bigr)^{T}H _{k}d^{k}-\sum _{j\in I_{*}}\lambda^{k}_{j}\nabla g_{j}^{k^{T}} \bigl(d ^{k}+\tilde{d}^{k} \bigr)+o \bigl( \bigl\Vert d^{k} \bigr\Vert ^{2} \bigr). \end{aligned}$$

Therefore, by (40) and Taylor expansion for $g_{j}(x^{k}+d ^{k})$ at point $x^{k}$, one yields

$$\begin{aligned} \nabla g_{j}^{k^{T}} \bigl(d^{k}+ \tilde{d}^{k} \bigr)=-g_{j}^{k}-\frac{1}{2} \bigl(d ^{k} \bigr)^{T}\nabla^{2}g_{j} \bigl(x^{k} \bigr)d^{k}+o \bigl( \bigl\Vert d^{k} \bigr\Vert ^{2} \bigr), \quad j\in I_{*}. \end{aligned}$$

This, together with (44), shows that

45 $$\begin{aligned} \nabla f_{\bar{\rho }} \bigl(x^{k} \bigr)^{T} \bigl(d^{k}+\tilde{d}^{k} \bigr)&= \bigl(d^{k} \bigr)^{T} \biggl(-H _{k}+\frac{1}{2}\sum _{j\in I_{*}}\lambda^{k}_{j}\nabla^{2}g _{j} \bigl(x^{k} \bigr) \biggr)d^{k} \\ &\quad {} +\sum_{j\in I_{*}}\lambda^{k}_{j}g_{j}^{k}+o \bigl( \bigl\Vert d^{k} \bigr\Vert ^{2} \bigr). \end{aligned}$$

On the other hand, the first relation of (39) gives

$$\begin{aligned} \lambda^{k}_{j}\nabla g_{j}^{k^{T}}d^{k}=- \bigl( \bigl(\lambda^{k}_{j} \bigr)^{2}/z ^{k}_{j} \bigr)g_{j}^{k}+o \bigl( \bigl\Vert d^{k} \bigr\Vert ^{2} \bigr), \quad j\in I_{*}. \end{aligned}$$

This, along with (43), shows that

46 $$\begin{aligned} \bigl(d^{k} \bigr)^{T}H_{k}d^{k}=- \nabla f_{\bar{\rho }} \bigl(x^{k} \bigr)^{T}d^{k}+ \sum_{j\in I_{*}}\frac{(\lambda^{k}_{j})^{2}}{z^{k}_{j}}g_{j}^{k}+o \bigl( \bigl\Vert d^{k} \bigr\Vert ^{2} \bigr). \end{aligned}$$

Again, substituting (45) into (41), one has

$$\begin{aligned} w_{k}&=\sum_{j\in I_{*}}\lambda^{k}_{j} g_{j}^{k}+\frac{1}{2} \bigl(d ^{k} \bigr)^{T} \bigl(\nabla^{2}_{xx}L_{\bar{\rho }} \bigl(x^{k},\lambda^{k} \bigr)-H_{k} \bigr)d ^{k} \\ &\quad {} -\frac{1}{2} \bigl(d^{k} \bigr)^{T}H_{k}d^{k}- \alpha \nabla f_{\bar{\rho }} \bigl(x^{k} \bigr)^{T}d ^{k}+o \bigl( \bigl\Vert d^{k} \bigr\Vert ^{2} \bigr). \end{aligned}$$

Therefore, substituting (46) into the relation above, we have

47 $$\begin{aligned} w_{k}&= \biggl(\frac{1}{2}-\alpha \biggr)\nabla f_{\bar{\rho }} \bigl(x^{k} \bigr)^{T}d^{k}+ \frac{1}{2} \bigl(d^{k} \bigr)^{T} \bigl(\nabla^{2}_{xx}L_{\bar{\rho }} \bigl(x^{k}, \lambda ^{k} \bigr)-H_{k} \bigr)d^{k} \\ &\quad {} +\sum_{j\in I_{*}}\lambda^{k}_{j} \biggl(1-\frac{\lambda^{k}_{j}}{2z _{j}^{k}} \biggr)g^{k}_{j}+o \bigl( \bigl\Vert d^{k} \bigr\Vert ^{2} \bigr). \end{aligned}$$

On the other hand, from the definition of the projection matrix $P_{k}$, we get

$$\begin{aligned} d^{k}=P_{k}d^{k}+d^{k}_{0}, \quad d^{k}_{0}=N_{k} \bigl(N_{k}^{T}N_{k} \bigr)^{-1}N _{k}^{T}d^{k}. \end{aligned}$$

Furthermore, in view of $\operatorname{SLE}(V_{k};1,\mu^{k})$, Theorem 2(iii) and the above division, one has

48 $$\begin{aligned} N_{k}^{T}d^{k}=Z_{k}^{-1} \bigl(\mu^{k}-\operatorname{diag} \bigl(g^{k}_{{I_{*}}} \bigr) \lambda ^{k}_{I_{*}} \bigr), \qquad d^{k}_{0}=o \bigl( \bigl\Vert d^{k} \bigr\Vert ^{2} \bigr)+O \bigl( \bigl\Vert g^{k}_{{I_{*}}} \bigr\Vert \bigr). \end{aligned}$$

Thus, relation (47), together with the relations above and H5, implies that

49 $$\begin{aligned} w_{k} =&\sum_{j\in I_{*}} \lambda^{k}_{j} \biggl(1-\frac{\lambda^{k} _{j}}{2z_{j}^{k}} \biggr)g_{j}^{k}+o \bigl( \bigl\Vert d^{k} \bigr\Vert ^{2} \bigr)+ \biggl( \frac{1}{2}-\alpha \biggr) \nabla f_{\bar{\rho }} \bigl(x^{k} \bigr)^{T}d^{k} \\ & {}+\frac{1}{2} \bigl(d^{k}_{0}+P_{k}d^{k} \bigr)^{T} \bigl(\nabla^{2}_{xx}L_{\bar{ \rho }} \bigl(x^{k},\lambda^{k} \bigr)-H_{k} \bigr) \bigl(d^{k}_{0}+P_{k}d^{k} \bigr) \\ =& \biggl(\frac{1}{2}-\alpha \biggr)\nabla f_{\bar{\rho }} \bigl(x^{k} \bigr)^{T}d^{k}+ \sum _{j\in I_{*}}\lambda^{k}_{j} \biggl(1- \frac{\lambda^{k}_{j}}{2z _{j}^{k}} \biggr) g_{j}^{k} +O \bigl( \bigl\Vert g^{k}_{{I_{*}}} \bigr\Vert \bigr)+o \bigl( \bigl\Vert d^{k} \bigr\Vert ^{2} \bigr). \end{aligned}$$

On the other hand, taking into account Lemma 6(ii), Lemma 7 and Theorem 2, one has (when $k\rightarrow \infty $)

50 $$ \lambda^{k}_{j}\rightarrow \lambda^{*}_{j}>0,\qquad \lambda^{k}_{j} \biggl(1-\frac{ \lambda^{k}_{j}}{2z^{k}_{j}} \biggr)\rightarrow \frac{\lambda^{*}_{j}}{2}>0,\quad \forall j\in I_{*}. $$

Further, relations (24), (13), (25), (23) and $\Vert d^{k} \Vert \sim \Vert \bar{d}^{k} \Vert $ as well as H2 yield

51 $$\begin{aligned} \nabla f_{\bar{\rho }} \bigl(x^{k} \bigr)^{T}d^{k} &\leq \theta \xi_{k}\leq \theta \nabla f_{\bar{\rho }} \bigl(x^{k} \bigr)^{T}\bar{d}^{k} \\ & =-\theta \bigl(\bar{d}^{k} \bigr)^{T}Q _{k} \bar{d}^{k}\leq \theta a \bigl\Vert \bar{d}^{k} \bigr\Vert ^{2} \\ & =-\theta a \bigl\Vert d^{k} \bigr\Vert ^{2}+o \bigl( \bigl\Vert d^{k} \bigr\Vert ^{2} \bigr). \end{aligned}$$

Therefore, for *k* large enough, relations (49)-(51) show that $w_{k}\leq (\alpha -\frac{1}{2})\theta a\Vert d^{k} \Vert ^{2}+o(\Vert d^{k} \Vert ^{2})\leq 0$. Thus, inequality (19) holds for $t=1$ and *k* large enough, and the entire proof of Theorem 3 is finished. □

Finally, based on Theorem 3, by similar analysis in [1, 18, 22] (for two-step superlinear convergence) and [21], Appendix A (for one-step superlinear convergence), we can prove the following rate of superlinear convergence.

### Theorem 4

*Suppose that* $\tilde{X}\neq\emptyset $ *and the hypotheses* H2-H5 *hold*. *If the boundary parameters* $\underline{\varepsilon}$ *and* *ε̅* *satisfy* (34), *then the proposed Algorithm* A *is two*-*step superlinearly convergent*, *i*.*e*., $\Vert x^{k+2}-x^{*} \Vert =o(\Vert x^{k}-x^{*} \Vert )$. *Moreover*, *if* H5 *is strengthened as* H5^+^, *then Algorithm* A *is one*-*step superlinearly convergent*, *i*.*e*., $\Vert x^{k+1}-x^{*} \Vert =o(\Vert x^{k}-x^{*} \Vert )$.

## Numerical experiments

In this section, to show the practical effectiveness of Algorithm A, we test 59 typical problems from [33]. The numerical experiments are implemented by using MATLAB R2013a, and on a PC with Inter(R) Core(TM) i5-4590 3.30 GHz CPU, 4.00 GB RAM. The details about the implementation are described as follows.

### Computing matrix $H_{k}$

During the process of iteration, to ensure the boundedness of $\{H_{k}\}$, by modifying the computing technique in [1] for the approximate Lagrangian Hessian, we introduce a slightly new computing method for the approximate Hessian matrix $H_{k}$ in Step 2 as follows from second order derivative information. Denote vector $\hat{z}^{k}$ and matrix $\mathcal{M}_{k}$ by

52 $$\begin{aligned} &\hat{z}^{k}= \bigl(z^{k}_{I^{\ell }}, z^{k}_{{I^{\imath }_{k}}}, 0_{{I ^{\imath }\setminus I^{\imath }_{k}}} \bigr), \end{aligned}$$

53 $$\begin{aligned} &\mathcal{M}_{k}=\nabla_{xx}^{2}L_{\rho_{k}} \bigl(x^{k},\hat{z}^{k} \bigr)- \sum _{j\in I}\frac{\hat{z}^{k}_{j}}{g_{j}^{k}}\nabla g_{j}^{k} \nabla g_{j}^{k^{T}}. \end{aligned}$$

Then compute the smallest eigenvalue $\vartheta_{\min }^{k}$ of matrix $\mathcal{M}_{k}$, and yield

54 $$\begin{aligned} \theta_{k}= \textstyle\begin{cases} 0, &\mbox{if } \vartheta_{\min }^{k}> \underline{\varepsilon}; \\ -\vartheta_{\min }^{k}+\underline{\varepsilon}, &\mbox{if } \vert \vartheta_{\min }^{k} \vert \leq \underline{\varepsilon}; \\ 2\vert \vartheta_{\min }^{k} \vert , &\mbox{otherwise}. \end{cases}\displaystyle \end{aligned}$$

Subsequently, compute matrix $H_{k}$ in Step 2 by

55 $$\begin{aligned} H_{k}= \textstyle\begin{cases} \nabla_{xx}^{2}L_{\rho_{k}}(x^{k},\hat{z}^{k})+\theta_{k} E_{n}, &\mbox{if } \rho_{k}\leq \overline{\varepsilon} \mbox{ and } \theta_{k} \leq \overline{\varepsilon}; \\ E_{n}, & \mbox{otherwise}, \end{cases}\displaystyle \end{aligned}$$

where the positive parameters $\underline{\varepsilon}$ and *ε̅* same as the ones in Algorithm A are sufficiently small and sufficiently large, respectively.

The sequence $\{H_{k}\}$ of matrices defined above possesses nice properties as follows.

#### Theorem 5

*Suppose that* $\tilde{X}\neq\emptyset $ *and assumptions* H3 *and* H4(i) *hold*. *Yield matrix* $H_{k}$ *in Step* 2 *by* (52)-(55). *If the sequence* $\{x^{k}\}$ *yielded by Algorithm* A *is bounded*, *then the following results hold*.

(i) *The sequence* $\{H_{k}\}$ *is bounded and satisfies the positive definite restriction* (27) *with constant* $a=\underline{\varepsilon}$, *so* H2 *holds*.
(ii) *In addition*, *assume that* H4(ii) *and* (34) *are satisfied*. *Then*, *for* *k* *large enough*, *matrix* $\mathcal{M}_{k}$ *is positive definite*, $\vartheta_{\min }^{k}>0$ *and* $\theta_{k}<\underline{ \varepsilon}$. *Therefore*, $H_{k}$ *is always yielded by the first case in* (55), *i*.*e*.,
  56 $$\begin{aligned} H_{k}=\nabla_{xx}^{2}L_{\rho_{k}} \bigl(x^{k},\hat{z}^{k} \bigr)+\theta_{k} E_{n}, \quad \textit{when k is large enough}. \end{aligned}$$

*Further*, *it follows that*

$$\begin{aligned} \lim_{k\rightarrow \infty } \bigl\Vert \nabla_{xx}^{2}L_{\bar{\rho }} \bigl(x^{k}, \lambda^{k} \bigr)-H_{k} \bigr\Vert =\lim_{k\rightarrow \infty }\theta_{k}\leq \underline{ \varepsilon} \approx 0 \end{aligned}$$

*when* $\underline{\varepsilon}$ *is sufficiently small*. *In this sense*, *we say assumption* $\mathrm{H5}^{+}$ *is almost satisfied*.

#### Proof

(i) By the boundedness of $\{(x^{k},\hat{z}^{k})\}$, in view of H4(i), the boundedness of $\{H_{k}\}$ follows immediately from (55). To show the second claim of part (i), it is sufficient to discuss the case $H_{k}\neq E_{n}$. For any $d\in R^{n}$, from (22) and (52)-(55), one has

57 $$ d^{T} Q_{k} d=d^{T} \biggl(H_{k}-\sum_{j\in I}\frac{\hat{z}^{k}_{j}}{g _{j}^{k}} \nabla g_{j}^{k}\nabla g_{j}^{k^{T}} \biggr) d = d^{T} (\mathcal{M} _{k} +\theta_{k} E_{n})d. $$

On the other hand, due to the symmetry of matrix $\mathcal{M}_{k}$, there exists a real orthogonal matrix $U_{k}$ such that $\mathcal{M} _{k}=U_{k} \operatorname{diag}(\vartheta^{k})U^{T}_{k}$, where $\vartheta^{k}=( \vartheta^{k}_{i})$ is the eigenvalue vector of $\mathcal{M}_{k}$. Therefore,

$$\begin{aligned} d^{T} \mathcal{M}_{k} d&=d^{T} U_{k} { \operatorname{diag}} \bigl(\vartheta^{k} \bigr) U_{k} ^{T} d= \bigl(U_{k}^{T} d \bigr)^{T} { \operatorname{diag}} \bigl( \vartheta^{k} \bigr) U_{k}^{T} d \\ &= \sum_{i=1}^{n} \bigl(u^{k}_{i} \bigr)^{2}\vartheta^{k}_{i} \geq \vartheta_{\min }^{k}\sum_{i=1}^{n} \bigl(u^{k}_{i} \bigr)^{2}= \vartheta_{\min }^{k} \bigl(U_{k}^{T} d \bigr)^{T} U_{k}^{T}d= \vartheta_{\min } ^{k}\Vert d \Vert ^{2}, \end{aligned}$$

where $u^{k}=(u^{k}_{i}, i=1,\dots,n)=U_{k}^{T} d$. This, along with (57) and (54), shows that

$$\begin{aligned} d^{T} Q_{k} d=d^{T}\mathcal{M}_{k} d+ \theta_{k}\Vert d \Vert ^{2}\geq \bigl( \vartheta_{\min }^{k}+ \theta_{k} \bigr)\Vert d \Vert ^{2}\geq \underline{\varepsilon} \Vert d \Vert ^{2}. \end{aligned}$$

So request (27) is satisfied with $a=\underline{\varepsilon}$.

(ii) First, under the given conditions, one knows that all the assumptions requested in Theorem 2 and Lemma 7 are satisfied. So, by Theorems 2 and Lemma 7, it follows that

58 $$ \begin{aligned} &I^{\imath }_{k}\equiv I^{\imath }\bigl(x^{*}\bigr), \qquad \rho_{k}\equiv \bar{\rho}, \bigl(x^{k},\hat{z}^{k}\bigr)\rightarrow \bigl(x^{*},\lambda^{*}\bigr), \\ &\nabla_{xx}^{2}L_{\bar{\rho }}\bigl(x^{k}, \hat{z}^{k}\bigr) \rightarrow \nabla _{xx}^{2}L_{\bar{\rho }} \bigl(x^{*},\lambda^{*}\bigr)=\nabla_{xx}^{2}L \bigl(x^{*}, \lambda '\bigr). \end{aligned} $$

Therefore, taking the above results and the SOSC in H4(ii) into account, it is not difficult to show that matrix $\mathcal{M}_{k}$ is positive definite when *k* is large enough, and this together with (54)-(55) and (58) further implies that the remaining claims in part (ii) hold. □

Based on Theorem 5, comparing with [1], the following remark is given.

#### Remark 11

The technique (52)-(55) yielding matrix $H_{k}$ is a modification of the one in [1], Section 5.1, and they are unlike in two points. First, the former introduced in this work can ensure the boundedness of $\{H_{k}\}$ (see Theorem 5(i)), which plays a key role in the analysis of global and superlinear convergence; especially, in ensuring the penalty parameter $\rho_{k}$ is increased at most finitely many times. However, the latter in [1], Section 5.1, cannot ensure the boundedness of the sequence $\{W_{k}\}$ yielded by [1], Section 5.1 (corresponds to $\{H_{k}\}$ in this paper) since this strict relies on the bounded property of $\{(\rho_{k},\theta_{k})\}$, and one of the necessary conditions for the boundedness of $\{(\rho_{k},\theta_{k}) \}$ is just the boundedness of $\{W_{k}\}$ (see the proof of [1], Lemma 4.1). Second, by introducing $\hat{z}^{k}$ in the computation technique (52)-(55) rather than $z^{k}$ (corresponds the one denoted in [1], Section 5.1), the assumption H5^+^ is almost satisfied (see Theorem 5(iii)). If one still uses $z^{k}$ rather that $\hat{z}^{k}$ in (52)-(55), then the second order approximate condition H5^+^ even H5 would be difficult to be satisfied since $z^{k}_{{I^{\imath }\setminus I^{\imath }(x ^{*})}}\rightarrow \underline{\varepsilon}e_{{I^{\imath }\setminus I^{\imath }(x^{*})}}>0=\lambda^{*}_{{I^{\imath }\setminus I^{\imath }(x^{*})}}$ (by Theorem 2(iv)). Of course, in view of $\lim_{k\rightarrow \infty }\Vert z^{k}-\hat{z}^{k} \Vert =\underline{\varepsilon}$ which is small enough, it can be thought that the numerical performances with $z^{k}$ and $\hat{z}^{k}$ should possess no distinct difference.

### Choices of parameters

The parameters in our numerical testing are chosen as follows:

$$\begin{aligned}& r=0.5, \qquad \alpha =0.45,\qquad \theta =0.99,\qquad \beta =0.5, \\& \sigma =0.8,\qquad \xi =2.5,\qquad \overline{\varepsilon}=10^{5}, \qquad \underline{\varepsilon}=10^{-5}, \\& \nu =3,\qquad \rho_{0}=p=1, \qquad \vartheta =2, \qquad M=100, \\& \gamma_{1}=0.1, \qquad \gamma_{2}= \gamma_{3}=0.01, \qquad z_{0}=(1,\ldots,1). \end{aligned}$$

#### Remark 12

Analysis for lower bound $\underline{\varepsilon}$ and upper bound *ε̅*

First, by Theorems 1 and 2, it is known that, in terms of global and strong convergence of Algorithm A, there is no additional request on the lower bound $\underline{\varepsilon}$ and upper bound *ε̅*, i.e., any two positive constants should be suitable. Second, if one considers the rate of convergence of Algorithm A, by Theorem 4, parameters $\underline{\varepsilon}$ and *ε̅* should be sufficiently small and sufficiently large, respectively. However, if the initial values of $\underline{\varepsilon}$ and *ε̅* are chosen too small and/or too large, the numerical performances should be unstable. An ideal approach is to decrease $\underline{\varepsilon}$ and increase *ε̅* based on values $\min \{z^{k}_{i}, i\in I\}$ and $\max \{z^{k}_{i}, i\in I\}$, respectively.

### Termination rules

During the process of iteration, the implementation is terminated successfully if one of the following two conditions is satisfied:

(i) $\Vert \Phi (x^{k},\lambda^{k}) \Vert <10^{-5}$; (ii) $\Vert \bar{d}^{k} \Vert <10^{-5}$ and $\max \{-\bar{\lambda }^{k}_{j},j\in I^{ \imath }\}< 10^{-5}$.

### Numerical reports

For the sake of comparing equally, the same initial points as in [33] should be selected. However, Algorithm A starts with a feasible interior point, namely, $x^{0} \in \tilde{X_{0}}$, and some initial points given in [33] do not satisfy this request. So, other initial points for these problems are selected and listed in Table 1.

**Table 1:** **Feasible initial interior points for testing problems**

**Prob.**	$\boldsymbol{x^{0}}$	**Prob**.	$\boldsymbol{x^{0}}$	**Prob.**	$\boldsymbol{x^{0}}$
HS6	(2,2)	HS32	(0.1,0.7,0.1)	HS63	(1,1,1)
HS7	(0,1)	HS39	(−1,−1,0,0)	HS73	(1,1,1,1)
HS8	(4,2)	HS40	(2,−1,0,1)	HS78	(−2,1.5,1,−1,−1)
HS25	(50,25,1.5)	HS42	(1,1,1,1)	HS79	(0,0,0,0,0)
HS26	(0.2,0.2,0.2)	HS52	(1,−0.5,−1,0,1)	HS80	(−2,−1.5,1,1,1)
HS27	(0,0,0)	HS53	(−6 2 2 2 2)	HS81	(−1.7,1,1.5,−0.8,−0.8)
HS28	(0,0,0)	HS60	(0,0,0)	HS107	(0.8 0.8 10 10 1 1 1 1 1)
HS111	(−1,−1,−1,−1,−1,−1,−1,−1,−1,−1)			HS114	(1,745 12,000 110 3,048 1,974 89.2 92.8 8 3.6 145)

The numerical results are reported and compared with the ones from [1] in Table 2, where the columns have the following meanings:

Prob.: the problem number given in [33];
Itr: the number of iterations;
Nf: the number of function evaluations for *f*;
N: the total number of function evaluations for $g_{j}$;
*ρ̄*: the final value of $\rho_{k}$;
Tcpu: the CPU time (seconds);
$f_{\mathrm{final}}$:
the objective function value at the final iterate.

**Table 2:** **Numerical experiment compared reports**

**Prob.**	***n***	$\boldsymbol{m_{e}}$	$\boldsymbol{m_{i}}$	**Algorithm ** **A** **in this paper**						**Algorithm from [** 1 **]**		
				**Itr**	**Nf**	**N**	$\boldsymbol{\bar{\rho}}$	$\boldsymbol{f_{\mathrm{final}}}$	**Tcpu**	**Itr**	$\boldsymbol{\bar{\rho }}$	$\boldsymbol{f_{\mathrm{final}}}$
HS1	2	0	1	28	73	70	1	1.7825e − 18	0.02	24	1	6.5782e − 27
HS3	2	0	1	6	7	8	1	2.3501e − 06	0.01	4	1	8.5023e − 09
HS4	2	0	2	7	13	29	1	2.6667e + 00	0.01	4	1	2.6667e + 00
HS5	2	0	4	5	13	47	1	−1.9132e + 00	0.01	6	1	−1.9132e + 00
HS6	2	1	0	9	364	718	1	2.4199e − 07	0.03	7	2	0.0000e + 00
HS7	2	1	0	8	15	28	32	−1.7320e + 00	0.01	9	2	−1.7321e + 00
HS8	2	2	0	9	16	59	8,192	−1.0000e + 00	0.01	14	1	−1.0000e + 00
HS9	2	1	0	18	34	66	8,192	−4.9985e − 01	0.02	10	1	−5.0000e + 01
HS12	2	0	1	9	19	39	1	−3.0000e + 01	0.01	5	1	−3.0000e + 01
HS24	2	0	5	16	29	179	1	−1.0000e + 00	0.02	14	1	−1.0000e + 00
HS25	3	0	6	1	1	6	1	9.4934e − 31	0.01	62	1	1.8185e − 16
HS26	3	1	0	16	76	142	2	1.6085e − 04	0.02	19	2	2.8430e − 12
HS27	3	1	0	28	484	939	4	3.9958e − 02	0.05	14	32	4.0000e − 02
HS28	3	1	0	11	38	71	1,024	7.5674e − 08	0.01	6	1	0.0000e + 00
HS29	3	0	1	11	24	53	1	−2.2627e + 01	0.01	8	1	−2.2627e + 01
HS30	3	0	7	7	10	63	1	1.0000e + 00	0.02	7	1	1.0000e + 00
HS32	3	1	4	19	33	166	128	9.8818e − 01	0.02	24	4	1.0000e + 00
HS33	3	0	6	15	20	189	1	−4.5178e + 00	0.02	29	1	−4.5858e + 00
HS34	3	0	8	10	15	104	1	−8.3403e − 01	0.02	30	1	−0.8340e + 00
HS36	3	0	7	10	15	144	1	−3.3000e + 03	0.02	10	1	−3.3000e + 03
HS37	3	0	8	12	19	200	1	−3.4560e + 03	0.02	7	1	−3.4560e + 03
HS38	4	0	8	73	153	1,218	1	1.9761e − 11	0.06	37	1	3.1594e − 24
HS39	4	2	0	11	19	63	1	2.5328e − 04	0.02	19	4	−1.0000e + 00
HS40	4	3	0	49	108	726	2	−2.5000e − 01	0.05	4	2	−2.500e + 00
HS42	4	2	0	36	70	290	1,024	1.3883e + 01	0.03	6	4	1.3858e + 01
HS43	4	0	3	12	29	73	1	−4.4000e + 01	0.02	9	1	−4.4000e + 01
HS46	5	2	0	101	234	735	1	1.3088e − 04	0.05	25	2	6.6616e − 12
HS47	5	3	0	21	54	276	1	2.0468e − 04	0.04	25	16	8.0322e − 14
HS48	5	2	0	21	55	202	2,048	3.1361e − 09	0.02	6	4	0.0000e + 00
HS49	5	2	0	51	87	276	64	1.1761e − 02	0.03	69	64	3.5161e − 12
HS50	5	3	0	50	200	1,065	128	9.3190e − 05	0.04	11	512	4.0725e − 17
HS51	5	3	0	29	132	722	256	2.2808e − 05	0.03	8	4	0.0000e + 00
HS52	5	3	0	31	45	225	256	5.2930e + 00	0.03	4	8	5.3266e + 00
HS53	5	3	10	36	69	1,694	256	4.0734e + 00	0.06	5	8	4.0930e + 00
HS56	7	4	0	21	43	2,482	4	−2.6183e + 00	0.06	12	4	−3.4560e + 00
HS57	2	0	3	34	53	141	1	2.8461e − 02	0.03	15	18	2.8460e − 02
HS60	3	1	6	18	43	574	1	3.2650e − 02	0.04	7	1	3.2568e − 02
HS61	3	2	0	16	255	986	256	−1.7195e + 02	0.03	44	128	−1.4365e + 02
HS62	3	1	6	8	19	153	1	−2.6273e + 04	0.02	5	1	−2.6273e + 04
HS63	3	2	3	15	27	200	1	9.6232e + 02	0.02	5	2	9.6172e + 02
HS66	3	0	8	15	42	249	1	5.1816e − 01	0.02	1,000^+^	1	5.1817e − 01
HS70	4	0	9	16	22	214	1	1.0085e − 02	0.03	22	1	1.7981e − 01
HS73	4	1	6	17	35	213	1	2.9896e + 01	0.03	16	1	2.9894e + 01
HS77	5	2	0	21	141	587	1	4.5981e − 01	0.06	13	1	2.4151e − 01
HS78	5	3	0	23	66	329	1	−2.9197e + 00	0.03	4	4	−2.9197e + 00
HS79	5	3	0	16	26	123	128	7.8681e − 02	0.02	7	2	7.8777e − 02
HS80	5	3	10	66	196	3,975	4	6.0149e − 02	0.14	6	2	5.3950e − 02
HS81	5	3	10	19	37	708	8	6.4109e − 02	0.05	9	8	5.3950e − 02
HS84	5	0	16	30	57	1,252	1	−5.2803e + 06	0.06	30	1	−5.2803e + 06
HS93	6	0	8	21	43	1,387	1	1.3629e + 02	0.04	12	1	1.3508e + 02
HS99	7	2	14	18	31	57	1	−8.3108e + 08	0.02	8	4	0.0000e + 00
HS100	7	0	4	8	22	86	1	6.8063e + 02	0.02	9	1	6.8063e + 02
HS107	9	6	8	41	67	1,086	1	1.3748e − 08	0.06	1,000^+^	8,192	5.0545e + 38
HS110	10	0	20	11	510	10,146	1	−4.3134e + 01	0.13	6	1	−4.5778e + 01
HS111	10	3	20	26	264	6,542	1,024	−5.8531e + 01	0.14	1,000^+^	1	−4.7760e + 01
HS112	10	3	10	6	11	199	2	−5.3197e + 01	0.02	11	1	−4.7761e + 01
HS113	10	0	8	21	48	519	1	2.4306e + 01	0.03	10	1	2.4306e + 01
HS114	10	3	28	11	136	4,474	16	−1.3407e + 03	0.13	39	256	−1.7688e + 03
HS118	15	0	59	34	51	2,554	1	6.6482e + 02	0.12	-	-	-

Same as the way of counting the number of iterations in [1], due to only a little change at the right side vector of SLE (10) in the loop between Step 3(i) and Step 3(ii), which leads to low computational cost, the number of this loop is not counted in the total number of iterations Itr.

From Table 2 it is clear that, for almost all test problems, the two algorithms (Algorithms A and the one in [1]) have the same optimal objective value. Relatively speaking, it also shows that Algorithm A is a promising one in terms of the CPU time, the number of function evaluations Nf and the total number of function evaluations N.

In particular, the following four performances are worth to be mentioned. First, for HS66, HS107 and HS111, the algorithm [1] yields the associated $f_{\mathrm{final}}$ after 1000 iterations for each problem, while Algorithm A needs only 15, 41 and 26 iterations, respectively. Second, for HS107, the two algorithms yield two large different final objective function values $f_{\mathrm{final}}$, namely, 3,748e−08 and 5.0545e+38. Third, for HS118 with the same dimension as HS117, Algorithm A has a good numerical performance, while it is not reported in [1]. Fourth, for HS54, HS75, HS85 and HS117, Algorithm A fails to produce an invertible coefficient matrix after some iterations, then it cannot obtain the optimal objective value, so they are not listed in Table 2.

For more clarity, we also give the output of Algorithm A for problem HS8 in Table 3. It is found from $\rho_{k}$-column of Table 3 that the penalty parameter needs to be increased one, two, four and six times at 2nd, 3rd, 4th and 5th iterations, respectively; and it can be fixed in the subsequent iterations.

**Table 3:** **Output of Algorithm** **A** **for problem HS8**

***k***	$\boldsymbol{\rho _{k}}$	$\boldsymbol{x^{k}}$	$\boldsymbol{f(x^{k})}$	$\boldsymbol{\Vert \bar{d}^{k} \Vert } $	$\boldsymbol{\Vert g^{k}_{\ell } \Vert } $
0	1	(4, 2)	−1.00000e+00		5.09902e+00
1	1	(4.56235, 1.91528)	−1.00000e+00	5.70399e−01	5.09902e+00
2	2	(4.60502, 1.91721)	−1.00000e+00	1.02139e−01	5.79231e−01
3	8	(4.60222, 1.94248)	−1.00000e+00	1.07010e−01	2.08008e−01
4	128	(4.60158, 1.95367)	−1.00000e+00	1.83034e−01	7.60755e−02
5	8,192	(4.60159, 1.95516)	−1.00000e+00	1.93245e−01	1.32456e−02
6	8,192	(4.60159, 1.95575)	−1.00000e+00	5.86992e−04	4.14795e−03
7	8,192	(4.60159, 1.95581)	−1.00000e+00	1.24840e−04	5.82513e−04
8	8,192	(4.60159, 1.95583)	−1.00000e+00	2.60839e−05	2.36077e−04
9	8,192	(4.60159, 1.95584)	−1.00000e+00	1.21999e−05	7.55776e−05

## Conclusions

In this paper, based on a simple and effective penalty parameter update rule and using the idea of primal-point interior method, a primal-dual interior point QP-free algorithm for nonlinear constrained optimization is proposed and analyzed. A ‘working set’ technique for estimating the active set is used in this work, then we need to solve only two or three reduced systems of linear equations with the same coefficient matrix at each iteration. Under suitable CQ and assumptions including a relaxed positive definite restriction on the Lagrangian Hessian estimate $H_{k}$, *but without the isolatedness of the stationary points*, the proposed algorithm is globally and superlinearly convergent. Moreover, a slightly new computation technique for $H_{k}$ based on second order derivative information is introduced such that the associated assumptions, i.e., the boundedness of $\{H_{k}\}$, the relaxed positive definiteness and the 1-sided projection second order approximation H5^+^, are all (almost) satisfied. The numerical experiments based on the proposed computation technique for $H_{k}$ show that the proposed algorithm is promising.

## Results and discussion

In this work, a new primal-dual interior point QP-free algorithm for nonlinear optimization with equality and inequality constraints is proposed. The global and superlinear convergence are analyzed. Some effective numerical results are reported. As further work, there are several interesting problems worthy of discussing. First, refer to [21], improve the algorithm such that it can start from an arbitrary initial point. Second, try to get rid of the strict complementarity condition. Third, apply the ideas in the paper to minimax optimization problems, engineering problems and so on.
